# Fecal Microbiota and Metabolome of Children with Autism and Pervasive Developmental Disorder Not Otherwise Specified 

**DOI:** 10.1371/journal.pone.0076993

**Published:** 2013-10-09

**Authors:** Maria De Angelis, Maria Piccolo, Lucia Vannini, Sonya Siragusa, Andrea De Giacomo, Diana Isabella Serrazzanetti, Fernanda Cristofori, Maria Elisabetta Guerzoni, Marco Gobbetti, Ruggiero Francavilla

**Affiliations:** 1 Department of Soil, Plant and Food Sciences, University of Bari Aldo Moro, Bari, Italy; 2 Inter-departmental Centre for Industrial Agri-Food Research, University of Cesena, Cesena, Italy; 3 Department of Agricultural and Food Sciences, University of Bologna, Bologna, Italy; 4 Child Neurological and Psychiatric Unit, Department of Neurological and Psychiatric Sciences, University of Bari Aldo Moro, Bari, Italy; 5 Department of Interdisciplinary Medicine, University of Bari Aldo Moro, Bari, Italy; Charité, Campus Benjamin Franklin, Germany

## Abstract

This study aimed at investigating the fecal microbiota and metabolome of children with Pervasive Developmental Disorder Not Otherwise Specified (PDD-NOS) and autism (AD) in comparison to healthy children (HC). Bacterial tag-encoded FLX-titanium amplicon pyrosequencing (bTEFAP) of the 16S rDNA and 16S rRNA analyses were carried out to determine total bacteria (16S rDNA) and metabolically active bacteria (16S rRNA), respectively. The main bacterial phyla (Firmicutes, Bacteroidetes, Fusobacteria and Verrucomicrobia) significantly (*P*<0.05) changed among the three groups of children. As estimated by rarefaction, Chao and Shannon diversity index, the highest microbial diversity was found in AD children. Based on 16S-rRNA and culture-dependent data, *Faecalibacterium* and *Ruminococcus* were present at the highest level in fecal samples of PDD-NOS and HC children. *Caloramator*, *Sarcina* and *Clostridium* genera were the highest in AD children. Compared to HC, the composition of Lachnospiraceae family also differed in PDD-NOS and, especially, AD children. Except for *Eubacterium siraeum*, the lowest level of Eubacteriaceae was found on fecal samples of AD children. The level of Bacteroidetes genera and some *Alistipes* and *Akkermansia* species were almost the highest in PDD-NOS or AD children as well as almost all the identified Sutterellaceae and Enterobacteriaceae were the highest in AD. Compared to HC children, *Bifidobacterium* species decreased in AD. As shown by Canonical Discriminant Analysis of Principal Coordinates, the levels of free amino acids and volatile organic compounds of fecal samples were markedly affected in PDD-NOS and, especially, AD children. If the gut microbiota differences among AD and PDD-NOS and HC children are one of the concomitant causes or the consequence of autism, they may have implications regarding specific diagnostic test, and/or for treatment and prevention.

## Introduction

Autism spectrum disorders (ASD) are complex neurodevelopmental dysfunctions, which are characterized by impairments of the social interaction and communication, as well as by restricted patterns of interest and repetitive behaviors [1]. ASD include autism (AD), Asperger’s Syndrome and Pervasive Developmental Disorder Not Otherwise Specified (PDD-NOS). Children with an ASD who lose skills (e.g., social interaction and communication) have become known as a subgroup called regressive autism or late onset. Regressive autism usually refers to a child where parents report an early history of normal development for 12-24 months which is followed by a loss of previously acquired skills. Individuals with ASD often suffer from gastrointestinal (GI) disorders (e.g., diarrhea, constipation, bloating and gastro-esophageal reflux) [2,3]. Epidemiology of ASD is increasing; its prevalence is estimated to be ca. 0.15% children for strict ASD [4] and 0.6-1% for broad ASD [5]. Research on ASD was mainly focused on genetic association but recent evidences suggest that other environmental factors may play a role in the disease [6,7]. Some reports highlighted that cognitive and social functions are somewhat improved in ASD patients, who were subjected to exclusion diet (e.g., gluten-free and/or casein-free diet) or treated with vancomycin [8,9]. Recently, other studies also reported that the GI microbiota is affected during AD pathogenesis [3,10–15]. 

The human GI microbiota is a complex consortium of 10^14^ microbes, whose collective genomes (microbiome) contain at least 100 times as many genes as our own eukaryote genome [16]. More than 10^3^ different species are capable of living in the human intestinal ecosystem [17]. Doubtless, GI microbiota has a key role on health and disease [15,18,19]. GI microbiota contributes to breakdown of dietary constituents, which are non-digestible in the upper gut [20], and is intimately involved in various and numerous aspects of the normal host physiology such as protection against pathogens, education of the immune system and modulation of the gastrointestinal development. Besides, microbes play a pivotal role or are the cause of several diseases [19]. The composition of the GI microbiota is mainly influenced by genetic factors [21], age [22] and diet [23,24]. 

Alterations of the composition of the GI microbiota are associated with inflammatory bowel diseases and allergic diseases [25–27]. Differences in the composition of the GI microbiota were associated with Type 1 and Type 2 diabetes [28], and celiac sprue [29,30]. Recently, a gut-brain-microbiota axis was coined, which described the interactions between these three systems [31]. Although interactions between the three systems are multifactorial and not yet completely defined, the vagus nerve works as a communication conduit between GI microbiota and brain [32]. Compared to healthy individuals, AD patients seemed to be characterized by higher numbers and/or species of *Clostridia* [33,34], Bacteroidetes [35], *Desulfovibrio* [36], *Sutterella* spp. [37] and by lower levels of Firmicutes [35] and Verrucomicrobia [38]. It was hypothesized that regressive autism is primarily caused by overgrowth of certain bacteria in the bowel of these children, in turn related to use of antimicrobial agents that suppress other elements of the normal bowel microbiota, permitting overgrowth of the resistant microorganisms [12]. The most commonly used antibiotics in these children are penicillins and cephalosporins and, at present, there is a significant incidence of bacterial resistance to these agents. Resistant bacteria that are involved in the regressive autism include various strains belonging to *Clostridia, Sutterella* and *Desulfovibrio* [12]. Potentially, an over-abundance of bacterial toxins might be involved in the AD disease [11,39]. The composition of *Lactobacillus* sp. and *Bifidobacterium* species differed between AD patients and healthy children [3,35,38]. Nevertheless, other data did not support consistent GI microbial abnormalities in AD children but only suggested that aberrations may be found in a minority of AD children [40]. Recent studies also indicated that AD patients had unbalanced metabolites at serum, fecal and urine levels [3,10,41,42]. It was hypothesized that qualitative and quantitative differences of the microbiota influenced the profile of volatile organic compounds (VOCs) of AD patients [3]. To the best of our knowledge, a more in depth characterization of the GI microbiota and related metabolome is strongly needed for AD patients, as well as the role of bacteria during AD development and treatment has to be highlighted [15].

This study aimed at comparing AD and PDD-NOS patients and healthy children (HC) based on fecal microbiota and metabolome. Total and active fecal microbiota was analyzed through an integrate approach of culture-dependent and -independent methods and metabolomic analyses. Bacterial tag-encoded FLX-titanium amplicon pyrosequencing (bTEFAP), gas-chromatography mass spectrometry/solid-phase microextraction (GC-MS/SPME) and Biochrom 30 series Amino Acid Analyser were carried out for genomic and metabolomic analyses, respectively.

## Materials and Methods

### Study design

This study was approved by the Institutional Review Board of Azienda Ospedaliero-Universitaria Consorziale Policlinico di Bari (Italy) and informed written consent was obtained from parents. The study was focused on 30 children (4 - 10 years of age; 14 male and 16 female); referred to the Child Neurological and Psychiatric Unit of Bari University Hospital for symptoms related to autistic spectrum disorders. In accordance with the DSM-IV-TR criteria, the children were grouped in ten PDD-NOS and AD. Three groups of children were included in the study: (i) ten PDD-NOS patients (children numbered: 1 - 10 PDD-NOS); (ii) ten AD patients (children numbered: 1 - 10 AD); and (iii) ten HC children without known diseases (children numbered: 1 - 10 HC). All PDD-NOS and AD children were assessed through direct free observations and diagnostic instruments: Autism Diagnostic Interview-Revised (ADI-R) [43], Autistic Diagnostic Observation Schedule (ADOS) [44] and Childhood Autism Rating Scale (CARS) [45]. All children in the HC group were brothers or sisters of PDD-NOS (1-5 HC) or AD (6-10 HC) children. Since each of these pairs of children belonged to the same family unit, the major dietary differences were excluded [29]. Exclusion criteria included the presence of neurological disorders of known etiology, major physical abnormalities, serious head injury [46], and presence of stomach/gut problems such as chronic diarrhea, constipation, gas, heartburn, bloating, etc. [3]. Children included in the study were not treated with antibiotics and/or functional foods (probiotics and/or prebiotics) for at least one month before sampling.

### Collection of fecal samples

Each child had fasted overnight, and fecal sample was collected in the morning pre-prandial. Each child provided three fecal samples over the time. After collection, feces, contained in sterile plastic box, were immediately mixed with RNA later (Sigma-Aldrich, St. Louis, MO, USA) (ca. 5 g, 1:2 w/v) or with Amies Transport medium (Oxoid LTD, Basingstoke, Hampshire, England) (ca. 15 g, 1:1 w/w) under anaerobic conditions (AnaeroGen, Oxoid LTD). Fecal samples suspended in RNA later were stored at -80°C for further DNA and RNA analyses. Samples diluted with Amies Transport medium were immediately subjected to culture-dependent (plate counts) and metabolome analyses. 

### DNA extraction from fecal samples

After homogenization in RNA later, fecal samples were mixed 1:1 with distilled water in sterile plastic pestle. The homogenate was subjected to mechanical disruption in a FastPrep^®^ instrument (BIO 101) and total DNA was extracted with a FastDNA^®^ Pro Soil-Direct Kit (MP Biomedicals, CA., USA), according to the manufacturer’s instructions. An aliquot of about 300 µL of each fecal sample was diluted in 1 mL of PBS-EDTA (phosphate buffer 0.01 M, pH 7.2, 0.01 M EDTA). After centrifugation (14,000 × *g* at 4°C for 5 min), the pellet was washed two times to decrease the content of PCR inhibitors. The resulting pellet was resuspended in 300 µL of PBS-EDTA and used for DNA extraction with a FastPrep. The product consisted of 50-100 µL of application-ready DNA. Quality and concentration of DNA extracts were determined in 1% agarose-0.5X TBE gels stained with Gel Red TM 10,000X (Biotium, Inc.) and by spectrophotometric measurements at 260, 280 and 230 nm using the NanoDrop^®^ ND-1000 Spectrophotometer (ThermoFisher Scientific Inc., MI., Italy).

### RNA extraction from fecal samples

An aliquot of ca. 200 mg of fecal sample diluted in RNA later was used for RNA extraction with the Stool total RNA purification kit (#49400 Norgen). Total RNA was treated with RNase-free DNase I (Roche, Almere, Netherlands; 10 U of DNase per 20 μg of RNA) for 20 min at room temperature. Quality and concentration of RNA extracts were determined using 1% agarose-0.5X TBE gels and by spectrophotometric measurements at 260, 280 and 230 nm using the NanoDrop^®^ ND-1000 Spectrophotometer. Total RNA extracted (ca. 2.5 µg) was transcribed to cDNA using random examers and the Tetro cDNA synthesis kit from Bioline (BIO-65043, Bioline), according to the manufacturer’s instructions [47]. 

### Bacterial tag-encoded FLX amplicon pyrosequencing (bTEFAP) and data analyses

For each child, the three DNA or cDNA samples corresponding to the three sampling were pooled and used for bTEFAP analysis. bTEFAP was performed by Research and Testing Laboratories (Lubbock, TX), according to standard laboratory procedures using a 454 FLX Sequencer (454 Life Sciences, Branford, CT, USA). Both DNA and cDNA were analyzed by bTEFAP. Primers forward 28F: GAGTTTGATCNTGGCTCAG and reverse 519R: GTNTTACNGCGGCKGCTG based upon the V1–V3 region (*Escherichia coli* position 27–519) of the 16 S rRNA gene were used [48]. The bTEFAP procedures were performed based upon RTL protocols http://www.researchandtesting.com (Research and Testing Laboratories, Lubbock, TX). Raw sequence data were screened, trimmed, and filtered with default settings using the QIIME pipeline version 1.4.0 (http://qiime.sourceforge.net). Chimeras were excluded by using the B2C2 (http://www.researchandtesting.com/B2C2.html) [49]. Sequences less than 250 bp were removed. FASTA sequences for each sample, without chimeras, were evaluated using BLASTn against a database derived from GenBank (http://ncbi.nlm.nih.gov) [50]. 

### Taxonomic Identification

The sequences were first clustered into OTU clusters with 100% identity (0% divergence) using USEARCH [51]. To determine the identities of bacteria, sequences were first queried using a distributed BLASTn .NET algorithm [50] against a database of high-quality 16S bacterial sequences derived from NCBI. Database sequences were characterized as high quality based upon the criteria originally described by Ribosomal Database Project (RDP, v10.28) [52]. Using a .NET and C# analysis pipeline, the resulting BLASTn outputs were compiled and validated using taxonomic distance methods, and data reduction analysis was performed as described previously [53]. Based upon the above BLASTn derived sequence identity percentage, the sequences were classified at the appropriate taxonomic levels based upon the following criteria. Sequences with identity scores, to well characterized database sequences, greater than 97% identity (<3% divergence) were resolved at the species level (Operational taxonomic units, OTUs), between 95% and 97% at the genus level, between 90% and 95% at the family and between 85% and 90% at the order level, 80 and 85% at the class and 77% to 80% at phyla. Any match below this identity level was discarded. The percentage of each bacterial identification (ID) was individually analyzed for each fecal sample, providing relative abundance information based upon relative numbers of reads within a given sample. Divergence of 3% and 5% is indicative of sequences differing at the species and genus level, respectively. The student’s t-test results seen in Table 1 indicate significantly higher diversity levels in PDD-NOS and AD when compared to HC. Alpha diversity (rarefaction, Good’s coverage, Chao1 richness and Shannon diversity indices) and beta diversity measures were calculated and plotted using QIIME [48,54,55]. Final datasets classified at the species and other relevant taxonomy levels were compiled into separate worksheets for the compositional analysis among the three groups of children [35]. Differences in microbial communities between PDD-NOS, AD and HC groups were also investigated using the phylogeny-based unweighted Unifrac distance metric [48]. 

**Table 1:** Biodiversity measures of total and metabolically active microbiota of Pervasive Developmental Disorder Not Otherwise Specified (PDD-NOS), autistic (AD), and healthy (HC) children.

**RFM**				**Chao**			**Shannon diversity index**		
**Genetic material**	**PDD-NOS**	**AD**	**HC**	**PDD-NOS**	**AD**	**HC**	**PDD-NOS**	**AD**	**HC**
**DNA**									
DNA (5%)	165	178	152	426	487	301	5.15	6.17	5.01
DNA (3%)	306	310	302	814	956	734	/	/	/
Group student’s t-test p-values									
(RFM 5%)		(RFM 3%)	/	(Chao 5%)	(Chao 3%)	/	(Shannon)		
HC vs PDD-NOS	0.165	0.152	/	0.064	0.070	/	0.090	/	/
HC vs AD	0.098	0.161	/	0.079	0.061	/	0.083	/	/
PDD-NOS vs AD	0.153	0.160	/	0.069	0.064	/	0.085	/	/
**RNA**									
RNA (5%)	104	108	95	317	320	286	5.14	5.32	4.25
RNA (3%)	233	291	214	691	869	642	/	/	/
Group student’s t-test p-values									
(RFM 5%)		(RFM 3%)	/	(Chao 5%)	(Chao 3%)	/	(Shannon)		
HC vs PDD-NOS	0.050	0.036	/	0.045	0.050	/	0.045	/	/
HC vs AD	0.048	0.011	/	0.044	0.039	/	0.040	/	/
PDD-NOS vs AD	0.025	0.043	/	0.055	0.041	/	0.052	/	/

Data are presented at 3% divergence level (corresponding roughly to the species level) and the 5% divergence level (corresponding roughly to the genus level) for rarefaction maximum predicted (RFM), Chao and Shannon diversity index.

### Enumeration of cultivable bacteria

Diluted fecal samples (20 g) were mixed with 80 mL sterilized physiological solution and homogenized. Counts of viable bacterial cell were carried out as described by Macfarlane et al. [56] The following selective media were used: Plate count agar (total facultative aerobes and anaerobes); MRS agar (lactobacilli and enterococci); *Bifidobacterium* agar modified (bifidobacteria) (Becton Dickinson France SA, Le Pont de Claix, France); M17 (*lactococci* and streptococci); Mannitol salt agar (staphylococci); Wilkins-Chalgren anaerobe agar (total anaerobes); Wilkins-Chalgren anaerobe agar plus GN selective supplements and sheep blood defibrinated (*Bacteroides*, *Porphyromonas* and *Prevotella*); Reinforced Clostridial Medium supplemented with 8 mg/L novobiocin, 8 mg/L colistin (*Clostridium*); MacConkey agar No2 (Enterobacteria); Rogosa agar plus 1.32 mL/L of glacial acetic acid (lactobacilli); GSP agar (Fluka) plus penicillin-G (60 g/L) (*Pseudomonas*, *Aeromonas*); and Slanetz and Bartley (Enterococci). Except for *Bifidobacterium* agar modified and GSP agar, all media were purchased by Oxoid Ltd (Hampshire, England).

### Concentration of free amino acids in feces

Total and individual free amino acids (FAAs) contained in fecal samples were analyzed by a Biochrom 30 series amino acid analyzer (Biochrom Ltd., Cambridge Science Park, England) with a sodium cation-exchange column (20 by 0.46 cm [inner diameter]). A mixture of amino acids at known concentrations (Sigma Chemical Co., Milan, Italy) was added with cysteic acid, methionine sulfoxide, methionine sulfone, tryptophan, ornithine, glutamic acid, and GABA and used as standard. Proteins and peptides in the samples were precipitated by addition of 5% (v/v) cold solid sulfosalicylic acid, holding the samples at 4°C for 1 h, and centrifuging them at 15,000 x *g* for 15 min. The supernatant was filtered through a 0.22-µm-pore-size filter and diluted, when necessary, with sodium citrate (0.2 M, pH 2.2) loading buffer. Amino acids were postcolumn derivatized with ninhydrin reagent and detected by absorbance at 440 (proline and hydroxyproline) or 570 (all the other amino acids) nm. 

### Gas-chromatography mass spectrometry-solid-phase microextraction (GC-MS/SPME) analysis of fecal volatile compounds

After preconditioning according to the manufacturer’s instructions, a carboxen-polydimethylsiloxane/Divinylbenzene coated fiber (CAR/PDMS Atable Flex 244) (85 μm) and a manual solid phase micro-extraction (SPME) holder (Supelco Inc., Bellefonte, PA, USA) were used. Before headspace sampling, the fiber was exposed to GC inlet for 5 min for thermal desorption at 250°C. Three grams of fecal sample were placed into 10 mL glass vials and added with 10 μL of 4-methyl-2-pentanol (final concentration of 33 mg/L), as the internal standard. Samples were then equilibrated for 10 min at 40°C. SPME fiber was exposed to each sample for 40 min. Both equilibration and absorption phases were carried out with stirring. The fiber was then inserted into the injection port of the gas chromatograph for 10 min of sample desorption. GC-MS analyses were carried out with an Agilent 7890A gas chromatograph (Agilent Technologies, Palo Alto, CA) coupled to an Agilent 5975C mass selective detector operating in electron impact mode (ionization voltage, 70 eV). A Varian Chromopack CP Wax 52 CB fused silica capillary column (length, 50 m; inside diameter, 0.32 mm x 1.2µm; Chromopack, Middelburg, The Netherlands) was used. The temperature program was 50°C for 1 min, followed by an increase at a rate of 4.5°C/min to 65°C, an increase at a rate of 10°C/min to 230°C, and then 230°C for 25 min. The injector, interface, and ion source temperatures were 250, 250, and 230°C, respectively. The mass-to-charge ratio interval was 30 to 350 Da at a rate of 2.9 scans per s. Injection was carried out with a split ratio 50:1 and helium (flow rate, 1 mL/min) was used as the carrier gas. Molecules were identified based on comparison of their retention times with those of pure compounds (Sigma-Aldrich, Milan, Italy). Identities were confirmed by searching mass spectra in the available databases (NIST, version 2005; Wiley, version 1996). All of the GC-MS raw files were converted to netCDF format via Chemstation (Agilent Technologies) and subsequently processed with the XCMS toolbox (http://metlin.scripps.edu/download/). XCMS software allows automatic and simultaneous retention time alignment, matched filtration, peak detection, and peak matching. The resulting table containing information such as peak index (retention time-m/z pair) and normalized peak area was exported into R (www.r-project.org) for subsequent statistical or multivariate analyses [57]. Quantitative data for the compounds identified were obtained by interpolation of the relative areas versus the internal standar area [58]. GC-MS/SPME data were organized into matrix and analyzed by Canonical discriminant Analysis of Principal coordinates [30].

### Statistical analysis

bTEFAP data (Unifrac distance metric and taxonomic abundance) were analyzed by Principal Component Analysis (PCA) to assess bacterial composition of samples [35,59] using a statistical software Statistica for Windows (Statistica 6.0 per Windows 1998, (StatSoft, Vigonza, Italia). Samples more similar to each other should appear closer together according to the respective axis reflecting the variation among all samples. This technique is useful in displaying clusters existing within data. The variables (features) are the relative bacterial composition in a sample at a particular taxonomic level [35]. In addition, Permut-MatrixEN software was used to identify clusters at the level of the PDD-NOS, AD and HC groups and taxa [60]. Culture dependent data and metabolomics data were obtained at least in triplicates. The analysis of variance (ANOVA) was carried out on transformed data followed by separation of means with Tukey’s HSD, using a statistical software Statistica for Windows (Statistica 6.0 per Windows 1998, (StatSoft). Letters indicate significant different groups (*P*<0.05) by Tukey’s test. Canonical discriminant Analysis of Principal coordinates (CAP) analysis was also carried out for GC-MS/SPME data [58]. The hypothesis of no significant difference in the multivariate location within groups was tested using the trace statistic based on 9999 permutations [58]. The correlation between metabolite concentrations and the number of predominant bacterial cells and genera was examined by linear regression analysis.

## Results

### Pyrosequencing analysis of the bacterial community of fecal samples

Pyrosequencing of 16S rDNA and rRNA was carried out to characterize total and metabolically active bacteria, respectively. Pyrosequencing of 30 sample sets yielded 201,048 and 120,121 bacterial 16S rDNA and 16S rRNA gene sequences, respectively. Pyrosequencing reads yielded an average of 5012 sequences (average length 432 bp) per sample. The bacterial community richness was analyzed by rarefaction curves, at 3 and 5% similarity levels, richness estimator (Chao1) and diversity index (Shannon) (Table 1). The average number of OTUs of 16S rRNA gene sequences found in fecal samples, at both 5 and 3% sequence divergence (species and genus levels, respectively), indicated significant difference between PDD-NOS, AD and HC. Compared to HC, the diversity of PDD-NOS and, especially, AD children, was higher. Almost similar results were found using three diversity and richness methods (including rarefaction, Chao1 and Shannon diversity index). As expected, higher levels of OTUs, Chao1 and Shannon diversity index were found for total compared to metabolically active bacteria.

**Figure 1:**
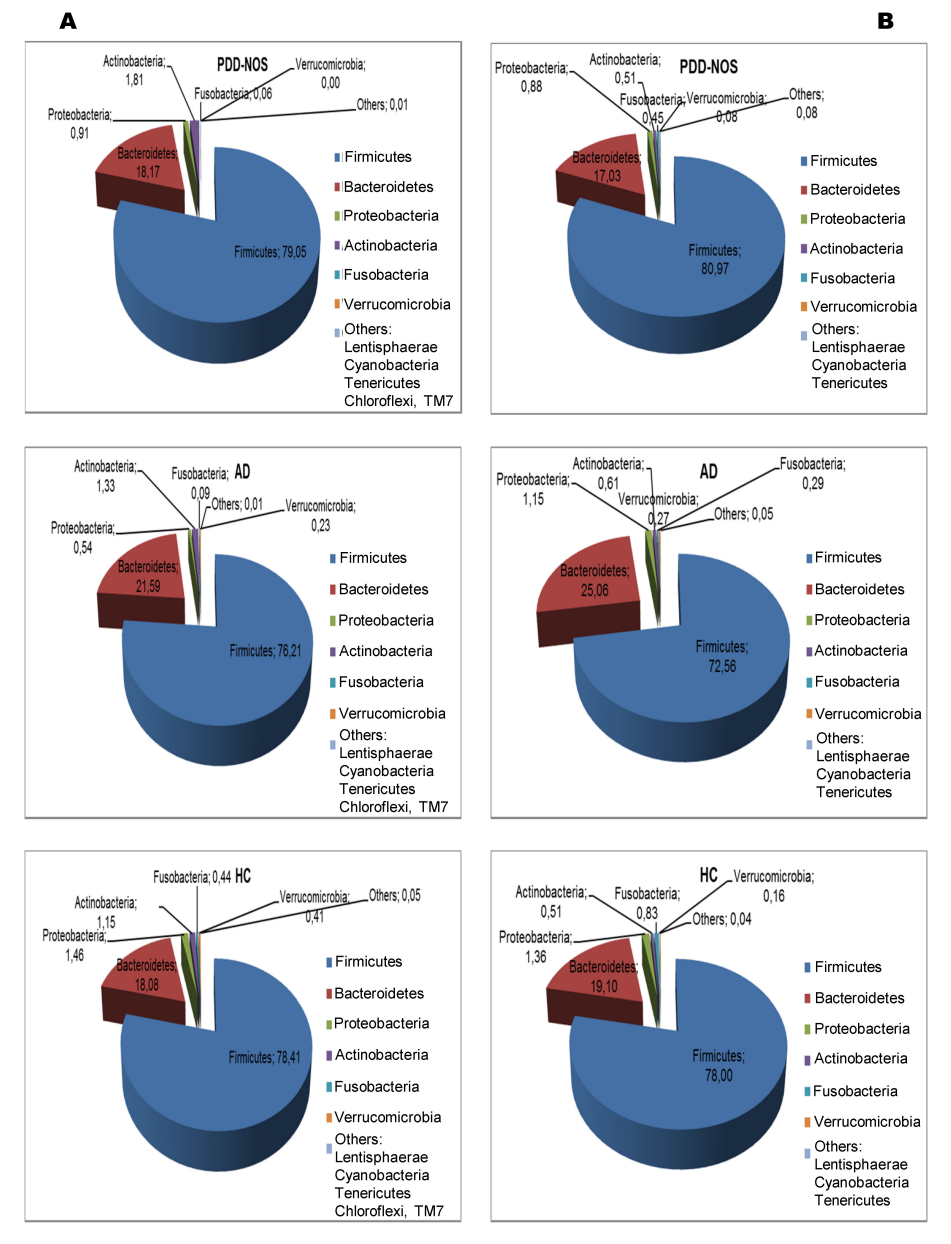
Total and active bacteria found in feces of children. Relative abundance (%) of total bacterial composition (16S-rDNA) (A) and metabolic active bacteria (16S-rRNA) (B) at the phylum level found in the fecal samples of Pervasive Developmental Disorder Not Otherwise Specified (PDD-NOS), autistic (AD) and healthy (HC) children.

**Figure 2:**
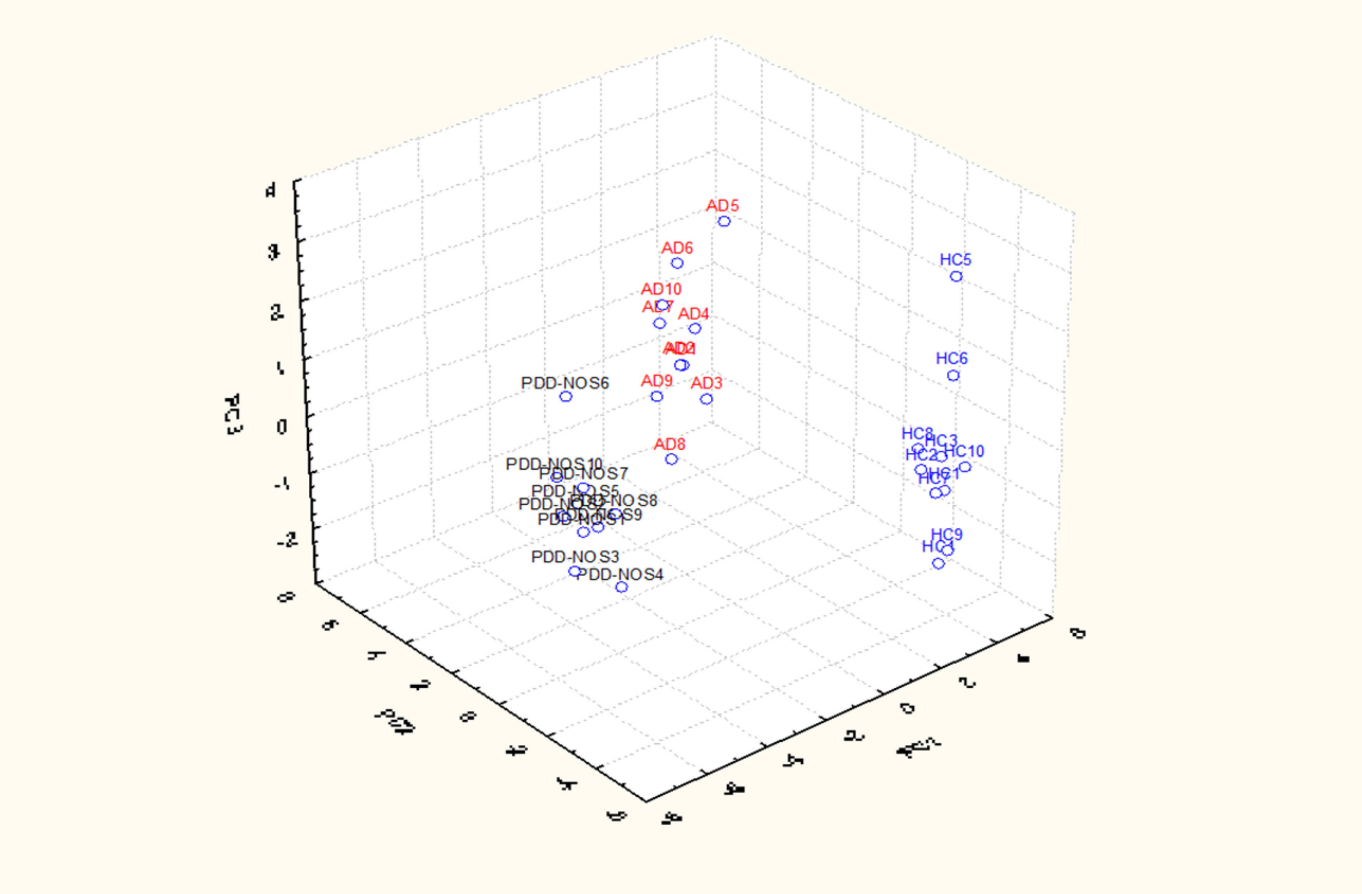
Principal component analysis (PCA) of active bacteria genera found in feces of children. Score plot of the three principal components (PC) after PCA of the total bacterial genera information (16S-rRNA) for Pervasive Developmental Disorder Not Otherwise Specified (PDD-NOS), autistic (AD) and healthy (HC) children. 1-10, number of fecal samples for each group of children.

**Figure 3:**
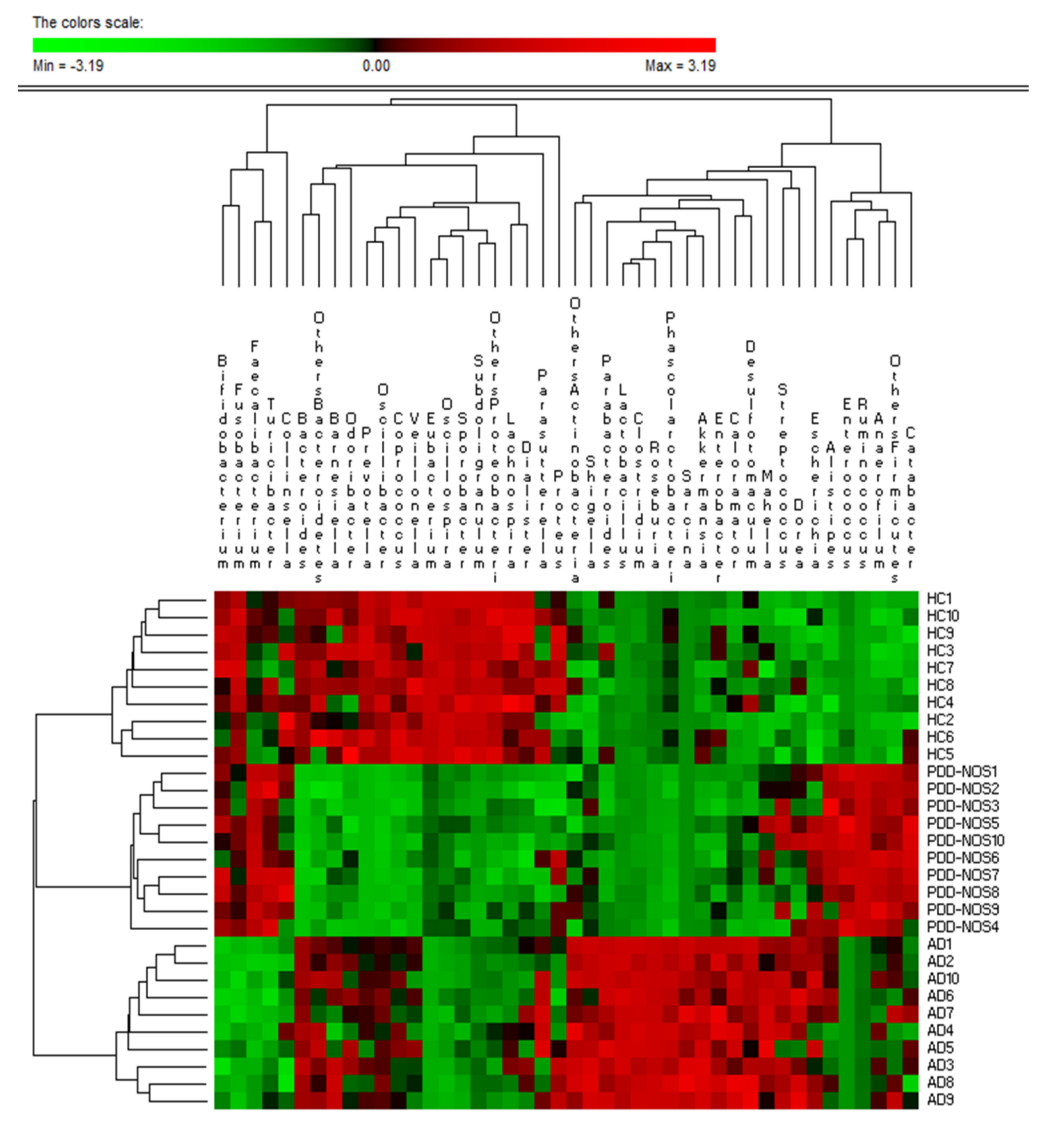
Permutation analysis of the active bacterial genera found in feces of children. Metabolically active bacterial genera composition found in Pervasive Developmental Disorder Not Otherwise Specified (PDD-NOS), autistic (AD) and healthy (HC) children. Double dendrogram representation of clustering was performed. The genera that showed values ​​less than 0.1% of the total metabolically active bacterial were grouped together on the same phylum (others Actinobacteria, others Bacteroidetes, others Firmicutes, others Proteobacteria). 1-10, number of fecal samples for each group of children.

Overall, a total of 11 (16S rDNA) and 9 (16S rRNA) bacterial phyla were identified in PDD-NOS, AD and HC children (Figure 1, Figure S1). TM7 and Chloroflexi phyla were not found as metabolically active bacteria. Firmicutes, Bacteroidetes, Proteobacteria, Actinobacteria, Fusobacteria and Verrucomicrobia represented more than 98% of all 16S rDNA and 16S rRNA gene sequences. Compared to HC, both total and active Firmicutes were lower in AD. No significant (*P*>0.05) and significant (*P*<0.05) differences were, respectively, found for total and metabolically active Firmicutes between PDD-NOS and HC. An opposite trend was found for Bacteroidetes, which showed the highest value in AD. Compared to HC, also Fusobacteria and Verrucomicrobia were found at lower levels in PDD-NOS and, especially, AD children (Figure 1). At phyla level, the major differences between total and metabolically active bacteria regarded Firmicutes (AD), Verrucomicrobia, TM7 and Chloroflexi (HC) (Figure S1). Using the genus composition data, 164 and 158 genera were found by 16S rDNA and rRNA analyses, respectively. Nevertheless, 45 and 40 genera, which were found at least into 80% of fecal samples of PDD-NOS, AD and/or HC, represented ca. 99 and 97% of the total or metabolically active bacteria, respectively. Unifrac distance metric and taxonomic abundance at genus level composition were further analyzed by PCA. As shown in the 3-D plot (Figure S2A), no systematic differences among 16S rDNA sequences associated to the three groups were found. On the contrary, fecal samples of PDD-NOS, AD and HC were clustered in different zone of the 3-D plot when 16S rRNA sequences were analyzed (Figure 2 and Table 2). This result was also confirmed through Permutation analyses (Figure 3). Based on this finding and considering that only metabolically active bacteria give a general picture of the microbial activities at intestinal level, further analyses were carried out only on 16S rRNA data. The main differences regarding the metabolically active bacteria are described below.

**Table 2:** Relative proportions of predominant metabolically active bacteria showing significant (P<0.05) differences between fecal samples of Pervasive Developmental Disorder Not Otherwise Specified (PDD-NOS), autistic (AD), and healthy (HC) children.

**Phylum/Family**	**Genus**	**Specie^a^**	**Avg (%) PDD-NOS**	**Avg (%) AD**	**Avg (%) HC**	**p-Val PDD-NOS vs HC**	**p-Val AD vs HC**	**p-Val PDD-NOS vs AD**
Firmicutes/Ruminococcaceae	*Faecalibacterium*	*Faecalibacterium prausnitzii*	17.82	11.37	12.59	0.002	0.04	0.008
		*Faecalibacterium* sp.	21.94	15.19	17.38	0.06	0.03	0.02
	*Ruminococcus*	*Ruminococcus* sp.	15.59	8.47	8.79	0.007	0.07	0.006
		*Ruminococcus obeum*	0.18	0.12	0.19	0.08	0.04	0.02
		*Ruminococcus gnavus*	0.03	0.13	0.11	0.009	0.08	0.007
	*Anaerofilum*	*Anaerofilum* sp.	0.46	0.24	0.07	0.001	0.003	0.008
	*Oscillospira*	*Oscillospira* sp.	0.91	0.77	1.06	0.004	0.002	0.04
	*Sporobacter*	*Sporobacter* sp.	0.18	0.11	0.17	0.07	0.05	0.04
		*Sporobacter termitidis*	0.18	0.22	0.39	0.02	0.04	0.06
	*Subdoligranulum*	*Subdoligranulum* sp.	0.63	0.79	1.78	0.009	0.006	0.05
Firmicutes/Clostridiaceae	*Caloramator*	*Caloramator* sp.	0.10	0.39	0.09	0.09	0.005	0.006
	*Sarcina*	*Sarcina ventriculi*	0.00	0.16	0.00	0.09	0.01	0.09
		*Sarcina* sp.	0.00	0.28	0.00	0.10	0.02	0.09
	*Clostridium*	*Clostridium* sp.	5.83	7.97	7.38	0.02	0.04	0.03
		*Clostridium orbiscindens*	0.04	0.11	0.05	0.09	0.03	0.02
		*Clostridium aminophilum*	0.07	0.34	0.08	0.09	0.03	0.02
		*Clostridium asparagiforme*	0.07	0.31	0.08	0.08	0.009	0.009
		*Clostridium bolteae*	0.32	1.16	1.06	0.008	0.06	0.009
		*Clostridium nexile*	0.13	0.15	0.21	0.04	0.04	0.07
		*Clostridium symbiosum*	0.05	0.18	0.05	0.09	0.03	0.04
		*Clostridium methylpentosum*	0.02	0.01	0.15	0.03	0.02	0.09
		*Clostridium bartlettii*	1.64	1.15	2.30	0.02	0.04	0.03
Firmicutes/Lachnospiraceae	*Roseburia*	*Roseburia* sp.	1.77	6.09	1.33	0.04	0.003	0.004
		*Roseburia faecis*	0.27	0.45	0.94	0.009	0.02	0.04
		*Roseburia inulinivorans*	0.39	2.66	0.14	0.04	0.008	0.009
		*Roseburia hominis*	0.02	0.09	0.11	0.04	0.06	0.05
		*Roseburia intestinalis*	0.18	0.31	0.50	0.03	0.04	0.04
	*Dorea*	*Dorea* sp.	0.52	0.63	0.35	0.04	0.03	0.06
	*Coprococcus*	*Coprococcus* sp.	0.04	0.12	0.17	0.04	0.05	0.04
		*Coprococcus eutactus*	0.04	0.22	0.25	0.02	0.07	0.03
	*Lachnospira*	*Lachnospira pectinoschiza*	0.09	0.14	0.20	0.02	0.05	0.05
Firmicutes/Eubacteriaceae	*Eubacterium*	*Eubacterium coprostanoligenes*	0.80	0.51	0.84	0.08	0.04	0.04
		*Eubacterium ventriosum*	0.20	0.08	0.25	0.07	0.01	0.02
		*Eubacterium* sp.	4.89	2.19	6.18	0.007	0.001	0.008
		*Eubacterium siraeum*	0.08	1.66	0.08	0.08	0.009	0.007
		*Eubacterium eligens*	0.08	0.07	0.66	0.02	0.03	0.07
	*Enterococcus*	*Enterococcus casseliflavus*	0.17	0.00	0.01	0.03	0.09	0.02
Firmicutes/Streptococcaceae	*Streptococcus*	*Streptococcus salivarius*	0.11	0.17	0.35	0.02	0.03	0.06
		*Streptococcus thermophilus*	0.24	0.38	0.12	0.02	0.009	0.04
		*Streptococcus* sp.	0.16	0.16	0.20	0.04	0.04	0.09
Firmicutes/Erysipelotrichaceae	*Turicibacter*	*Turicibacter* sp.	0.06	0.07	0.53	0.007	0.006	0.08
		*Turicibacter sanguinis*	0.33	0.18	0.13	0.03	0.07	0.03
Bacteroidetes/Bacteroidaceae	*Bacteroides*	*Bacteroides* sp.	9.67	14.56	12.66	0.03	0.04	0.02
		*Bacteroides uniformis*	0.70	1.35	3.12	0.01	0.02	0.03
		*Bacteroides fragilis*	0.16	0.61	0.04	0.02	0.008	0.009
		*Bacteroides massiliensis*	0.50	0.07	0.18	0.02	0.03	0.009
		*Bacteroides faecis*	0.00	0.33	0.05	0.05	0.02	0.01
		*Bacteroides ovatus*	0.07	0.12	0.16	0.02	0.06	0.04
		*Bacteroides vulgatus*	0.53	1.17	0.80	0.03	0.007	0.005
		*Bacteroides intestinalis*	0.00	0.79	0.04	0.06	0.03	0.02
		*Bacteroides coprocola*	1.29	0.00	0.35	0.01	0.03	0.008
Bacteroidetes/Porphyromonadaceae	*Barnesiella*	*Barnesiella intestinihominis*	0.25	0.74	0.54	0.02	0.04	0.008
	*Odoribacter*	*Odoribacter splanchnicus*	0.11	0.21	0.17	0.06	0.05	0.04
	*Parabacteroides*	*Parabacteroides* sp.	0.03	0.12	0.11	0.03	0.08	0.04
Bacteroidetes/Prevotellaceae	*Prevotella*	*Prevotella* sp.	0.00	0.37	0.89	0.01	0.02	0.01
		*Prevotella copri*	0.00	0.54	0.37	0.009	0.04	0.006
Bacteroidetes/Rikenellaceae	*Alistipes*	*Alistipes* sp.	0.47	0.29	0.27	0.02	0.07	0.03
		*Alistipes onderdonkii*	0.48	0.01	0.19	0.03	0.04	0.01
		*Alistipes shahii*	0.42	0.25	0.17	0.03	0.04	0.04
		*Alistipes indistinctus*	0.02	0.14	0.02	0.09	0.03	0.02
		*Alistipes putredinis*	1.80	2.03	1.57	0.04	0.01	0.03
Proteobacteria/Sutterellaceae	*Parasutterella*	*Parasutterella excrementihominis*	0.09	0.15	0.10	0.09	0.04	0.04
Proteobacteria/Enterobacteriaceae	*Enterobacter*	*Enterobacter hormaechei*	0.04	0.18	0.06	0.06	0.03	0.03
	*Escherichia*	*Escherichia coli*	0.09	0.06	0.16	0.04	0.03	0.06
	*Shigella*	*Shigella* sp.	0.08	0.18	0.09	0.08	0.03	0.02
Actinobacteria/Bifidobacteriaceae	*Bifidobacterium*	*Bifidobacterium* sp.	0.31	0.10	0.31	0.10	0.03	0.03
		*Bifidobacterium adolescentis*	0.28	0.09	0.23	0.06	0.01	0.009
		*Bifidobacterium catenulatum*	0.21	0.08	0.11	0.04	0.06	0.03
Actinobacteria/Coriobacteriaceae	*Collinsella*	*Collinsella* sp.	0.15	0.01	0.06	0.03	0.06	0.02
Fusobacteria/Fusobacteriaceae	*Fusobacterium*	*Fusobacterium* sp.	0.45	0.29	0.79	0.03	0.009	0.03
Verrucomicrobia/Verrucomicrobiaceae	*Akkermansia*	*Akkermansia muciniphila*	0.08	0.27	0.10	0.06	0.03	0.02

On average, Clostridia class was ca. 69.4 - 70.8% (AD and HC) and 78.4% (PDD-NOS) of the Firmicutes phylum, respectively. Ruminococcaceae, Clostridiaceae, Lachnospiraceae and Eubacteriaceae were the most commonly retrieved families of Clostridia. *Faecalibacterium* was the main genus (ca. 40.0, 27.0 and 33.0% for PDD-NOS, AD and HC, respectively) of the Ruminococcaceae family (data not shown). *Faecalibacterium prausnitzii*, a high producer of butyrate, was identified in all the samples (Table 2). One frequently recruited genus was *Ruminococcus*, which showed the highest number of reads in PDD-NOS. *Anaerofilum*, *Oscillospira*, *Sporobacter* and *Subdoligranulum* were identified at low frequencies. Except for *Anaerofilum*, all the other genera were the lowest in AD (Table 2, Figure 3). Other Clostridia related genera (*Caloramator* and *Sarcina*) were the highest in AD. Similar data were found for the *Clostridium* genus. *Clostridium nexile* (cluster XIVa), *Clostridium methylpentosum* (cluster IV) and *Clostridium bartlettii* (cluster XI) were the highest in HC. *Roseburia* was the dominant genus of the Lachnospiraceae family, with *Roseburia* sp. and *Roseburia inulinivorans* as the most frequently identified reads in AD. Quantitatively minor species (*Roseburia faecis* and *Roseburia intestinalis*) were the highest in HC. *Dorea* sp. was also found at the highest levels in PDD-NOS and AD as well as other minor Lachnospiraceae genera (*Coprococcus* and *Lachnospira*) were the lowest in PDD-NOS. Overall, the highest number of *Eubacterium* (4.89, 2.19 and 6.18% for PDD-NOS, AD and HC, respectively) was not attributed to a species (*Eubacterium* sp.). 

*Enterococcus* and *Streptococcus* were the dominant genera of the Bacilli class (Table 2, Figure 3). Compared to PDD-NOS and HC, *Enterococcus* species were lower in AD. Within Erysipelotrichi class, *Turicibacter sanguinis* and *Turicibacter* sp. were commonly identified (Table 2, Figure 3). *T. sanguinis* was found at the highest level in PDD-NOS and AD. On the contrary, *Turicibacter* sp was the highest in HC (Table 2). 

Reads within the Bacteroidaceae family (Bacteroidetes phylum) were attributed to the *Bacteroides* genus, with the highest percentage, which was not attributed at species level (9.67, 14.56 and 12.66% for PDD-NOS, AD and HC). Except for *Bacteroides uniformis* and *Bacteroides ovatus*, the highest level of species was found in PDD-NOS and, especially, AD. The species *Barnesiella intestinihominis*, *Odoribacter splanchnicus* and *Parabacteroides* sp were identified within Porphyromonadaceae (Table 2). All were the highest in AD. Reads related to Prevotellaceae were found at the highest levels in AD (*Prevotella copri* and *Prevotella oris*) and HC (*Prevotella* sp.). *Alistipes* species, belonging to Rikenellaceae family, were highest in AD and, especially, PDD-NOS.

Proteobacteria phylum comprised reads from Betaproteobacteria and Gammaproteobacteria classes. Within Betaproteobacteria, *Parasutterella excrementihominis* belonging to Sutterellaceae family, was the highest in AD (Table 2). Compared to HC, higher levels of Enterobacteriaceae genera and species were found in AD (Table 2, Figure 3). The only exception was *Escherichia coli*. 

*Bifidobacterium* and *Collinsella* were the dominant genera of the Actinobacteria (Table 2, Figure 3). Except for *Collinsella aerofaciens*, all the other species were the lowest in AD. *Fusobacterium* and *Akkermansia* were the only genera found within Fusobacteria and Verrucomicrobia phyla in almost all samples. Compared to HC, a lower level of *Fusobacterium* was found in PDD-NOS and, especially, AD (Table 2, Figure 3). On the contrary, *Akkermansia muciniphila* was the highest in AD.

### Enumeration of cultivable bacteria

Relatively selective media were used to enumerate cultivable cells of the main microbial groups (Table 3). No statistical difference (*P*>0.05) was found between PDD-NOS, AD and HC for total microbes. Total anaerobes were the highest in HC. Compared to HC, median values of presumptive *Clostridium* were higher in AD. The median values of presumptive *Enterococcus*, *Lactobacillus*, *Streptococcus, Lactococcus* and *Staphylococcus* of AD were lower than those found for HC. Compared to HC, significantly (*P*<0.05) higher counts of presumptive *Bacteroides*, *Porphyromonas* and *Prevotella*, *Pseudomonas*, *Aeromonas* and *Enterobacteria* were found in fecal samples of AD. Compared to HC and PDD-NOS, the number of presumptive *Bifidobacteria* significantly (*P*<0.05) decreased in AD children.

**Table 3:** Median values and range of cultivable cells (log CFU/g) of the main microbial groups in the fecal samples of Pervasive Developmental Disorder Not Otherwise Specified (PDD-NOS), autistic (AD), and healthy (HC) children.

**Microbial group**	**PDD-NOS**	**AD**	**HC**
Heterotrophic aerobic and anaerobic bacteria	7.48^a^ (5.78-8.88)	7.33^b^ (5.63-9.30)	7.47^a^ (6.40-8.79)
Total anaerobes	8.51^c^ (6.21-9.50)	8.78^b^ (7.09-9.54)	9.21^a^ (7.70-9.86)
*Clostridium*	5.24^b^ (3.06-8.00)	6.10^a^ (2.30-7.45)	4.74^c^ (0-6.70)
*Enterococcus* and *Lactobacillus*	7.04^b^ (5.69-8.32)	6.19^c^ (3.99-9.25)	7.24^a^ (6.23-8.33)
*Streptococcus* and *Lactococcus*	7.76^a^ (6.80-9.59)	7.06^b^ (4.24-9.21)	7.82^a^ (6.53-9.36)
*Staphylococcus*	7.01^a^ (5.47-8.48)	6.20^c^ (3.56-9.07)	6.77^b^ (4.04-8.55)
*Bacteroides*, *Porphyromonas* and *Prevotella*	5.52^b^ (3.74-7.57)	5.98^a^ (2.36-6.65)	4.92^c^ (2.60-7.12)
Enterobacteriaceae	6.82^a^ (5.30-8.00)	6.47^b^ (3.59-7.89)	6.03^c^ (5.83-8.00)
*Pseudomonas*, *Aeromonas*	4.83^b^ (2.54-8.34)	5.72^a^ (2.93-7.44)	4.99^b^ (0-7.28)
*Bifidobacterium*	8.00^a^ (6.65-9.58)	7.45^b^ (6.61-8.34)	8.09^a^ (5.58-9.15)

Data are the means of three independent experiments (n = 3) for each infant. ^a, b, c^ Values within a row with different superscript letters are significantly different (p < 0.05).

### Free amino acids (FAA) and volatile organic compounds (VOC) profiling of feces

**Figure 4:**
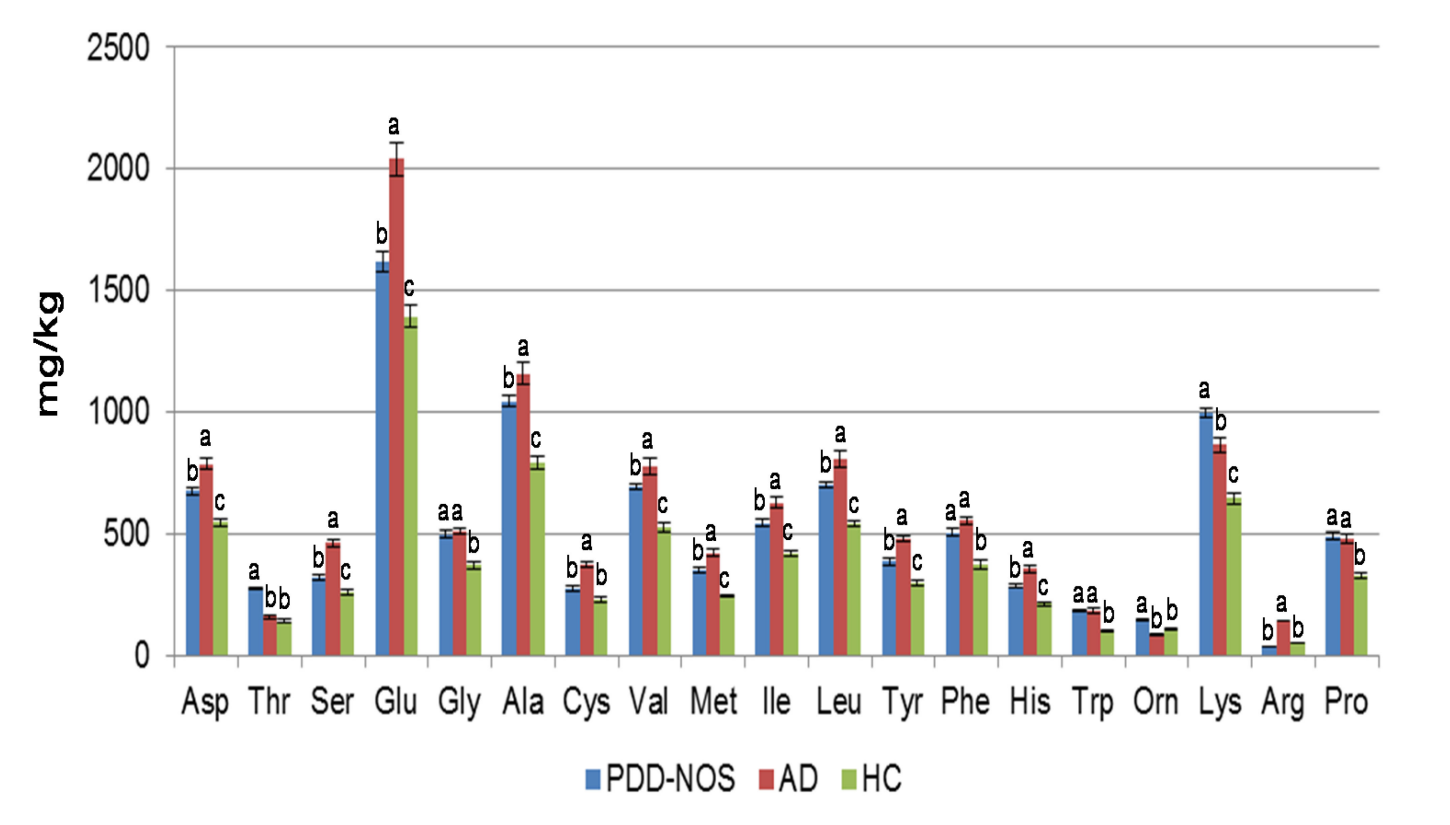
Fecal levels of free amino acids (FAA) in children. Concentration (mg/kg) of FAA found in fecal samples of Pervasive Developmental Disorder Not Otherwise Specified (PDD-NOS), autistic (AD) and healthy (HC) children. Data are the means of three independent experiments and standard deviations, performed in duplicate (n=6).

AD fecal samples contained the highest (*P*<0.05) level of total FAA (ca. 11299 vs. 7616 and 10059 mg/kg of feces for HC and PDD-NOS, respectively). Glu, Ala, Asp and Lys were the dominant FAA (Figure 4). In particular, Asp, Ser, Glu, Gly, Ala, Val, Ile, Phe, His, Tpr, Lys and Pro were found at the highest levels in PDD-NOS and, especially, AD. Besides, γ-amino-butyric acid (GABA), synthesized through decarboxylation of Glu, was the lowest in PDD-NOS fecal samples (ca. 168, vs. 252 and 244 mg/kg of feces for HC and AD, respectively). The concentration of ammonia (NH_3_) ranged from ca. 2045 (PDD-NOS) to 5726 (AD) mg/kg of fecal samples (data not shown). 

**Figure 5:**
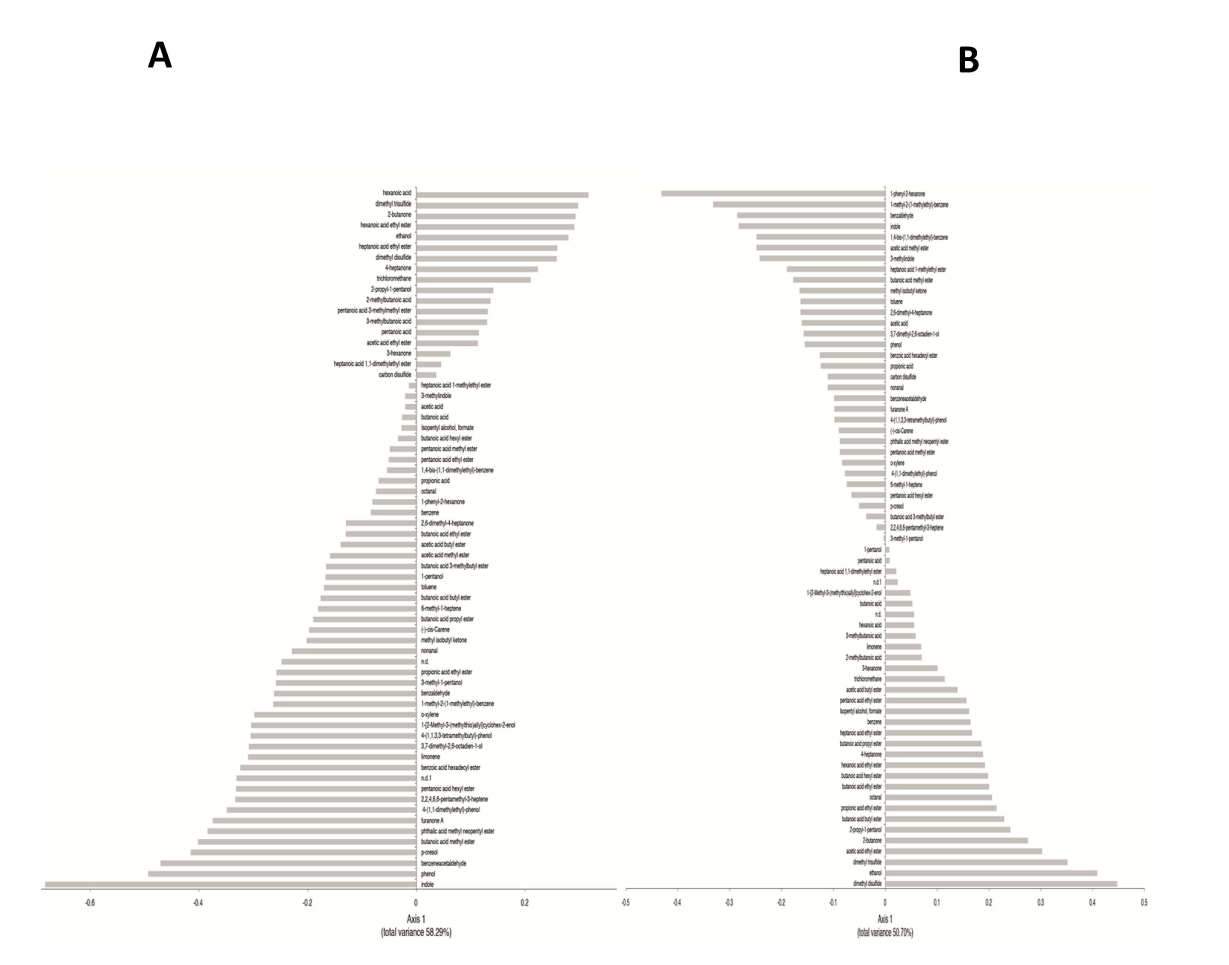
Canonical Discriminant Analysis of Principal Coordinates (CAP) of volatile organic metabolites in feces of children. Loading coefficient plots of the volatile organic compounds from fecal samples of Pervasive Developmental Disorder Not Otherwise Specified (PDD-NOS) (A), autistic (AD) (B) and healthy (HC) children. Compounds significantly associated with the feces of PDD-NOS children (negative axis) or HC children (positive axis) Data are the means of three independent experiments (n = 3).

**Figure 6:**
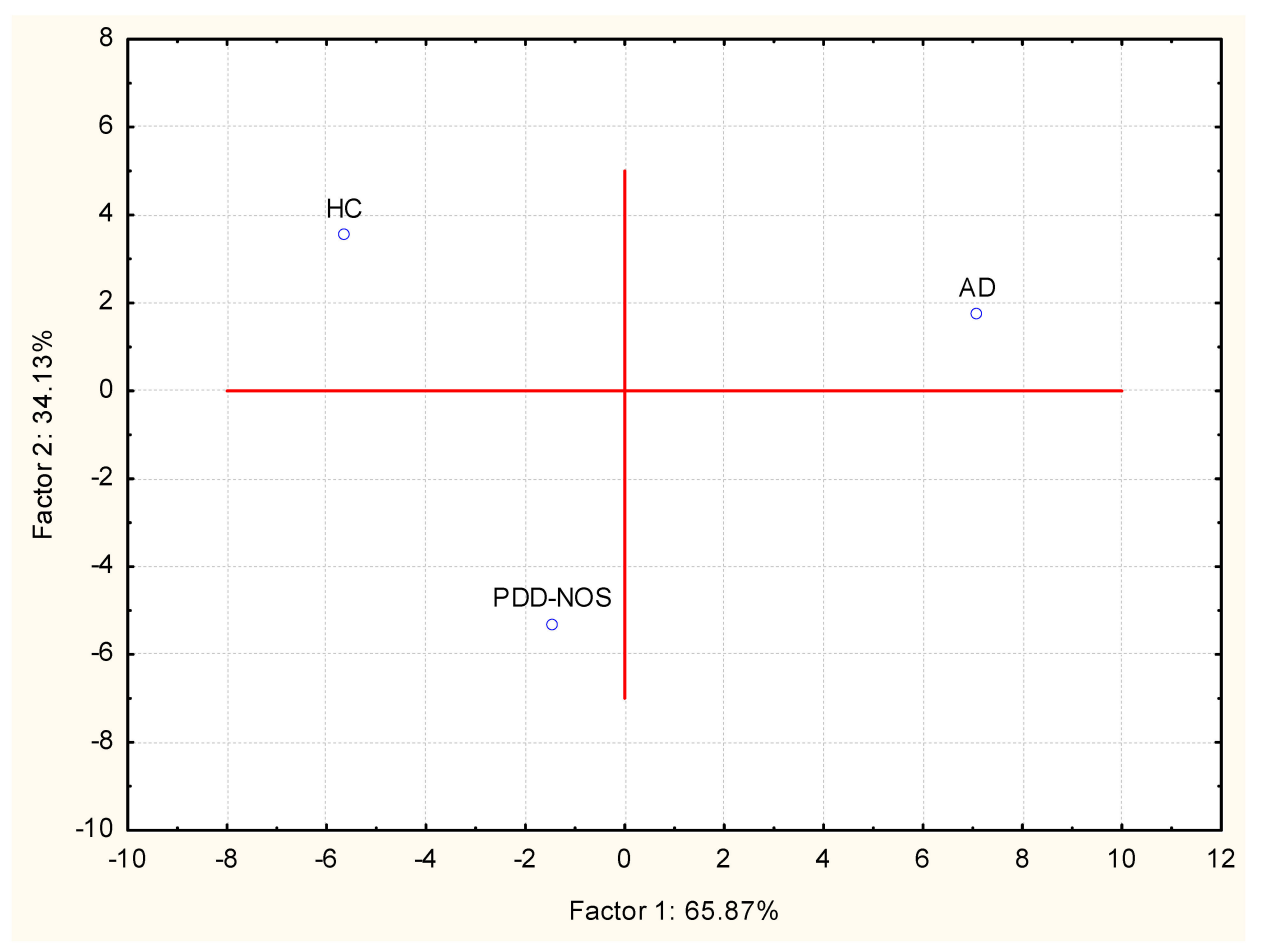
Principal component analysis (PCA) of volatile organic metabolites found in feces of children. Score plots of the two principal components (PC) after PCA of volatile organic metabolites found in fecal samples of Pervasive Developmental Disorder Not Otherwise Specified (PDD-NOS), autistic (AD) and healthy (HC) children. All the variables used were listed in Table S1.

VOC (82 compounds) were identified from fecal samples and grouped according to chemical classes: alcohols (13 compounds identified), aldehydes (4), esters (27), sulphur compounds (3), hydrocarbons (13), ketones (6), short and medium chain fatty acids (SCFA) (9), terpens (4), indoles (2) and furanones (1) (Table S1). Overall, the content of the various metabolites largely varied within the same group. GC-MS/SPME data were analyzed by a multivariate statistical approaches (Canonical discriminant Analysis of Principal coordinates, CAP and Principal Component Analysis, PCA). The result of the CAP analysis is shown in Figure 5A-B. Compounds appearing with negative values were those significantly (*P*<0.05) associated to PDD-NOS or AD, while those on the positive axis were significantly (*P*<0.05) associated to HC. The average values of total alcohols and phenols differed (*P*<0.05) between the three groups. Some compounds (ethanol, 2-propyl-1-pentanol and 1-pentanol) were found at significantly (*P*<0.05) higher levels in HC than in PDD-NOS and, especially, AD (Table S1, Figure 5A-B). Compared to HC, 3,7-dimethyl-2,6-octadien-1-ol, phenol, 4-(1,1,3,3-tetramethylbutyl)-phenol, p-cresol were higher in PDD-NOS and AD. Besides, 3-methyl-1-pentanol increased in PDD-NOS. Within aldehydes, octanal was found at the highest level in PDD-NOS as well as benzeneacetaldehyde and benzaldehyde were the highest in PDD-NOS and AD. The average value of esters was the lowest in AD. Nevertheless, the levels of some methyl esters (e.g., acetic acid methyl ester, pentanoic acid methyl ester, and heptanoic acid 1-methylethyl ester) were the highest in AD. Sulfur compounds also differed between PDD-NOS, AD and HC. The highest average value of hydrocarbons was found in AD. Nevertheless, the highest levels of benzene, trichloromethane and isopentyl alcohol, formate and 2,2,4,6,6-pentamethyl-3-heptene, 6-methyl-1-heptene were found in HC and/or PDD-NOS. With a few exceptions, ketones were found at highest levels in fecal samples of HC. The average value of total short and medium chain fatty acids (SCFAs) was significantly (*P*<0.05) higher in HC than in PDD-NOS and, especially, AD. Major differences were found for pentanoic and hexanoic acids. On the contrary, acetic and propionic acids were found at the highest levels in AD and PDD-NOS. Overall, fecal samples of PDD-NOS showed the highest levels of terpenes. The level of indoles (indole and 3-methylindole) were significantly (*P*<0.05) lower in fecal samples of HC compared those of PDD-NOS and, especially, AD. Differences on the metabolomes of PDD-NOS, AD and HC were highlighted through PCA analysis (Figure 6). According to the main VOC composition, the three groups of children were separated. Samples from PDD-NOS and HC children were more similar, while the samples of AD and HC children showed the lowest similarity. 

### Correlation between metabolically active bacteria and metabolomic data

The intestinal microbiota is responsible for several metabolic activities, which mainly occur at the colon level. A positive correlation was found between some metabolically active bacteria and metabolites that were found in fecal samples. A positive correlation (1.0; *P*<0.05) was found between the level of *Clostridium* species and the amount of methyl esters (butanoic acid methyl ester, acetic acid methyl ester and pentanoic acid methyl ester) and indoles. *Faecalibacterium* and *Ruminococcus* and *Bifidobacterium* genera were positively correlated (1.0; *P*<0.05) with total SCFA. *Bacteroides* genus was correlated (1.0; *P*<0.05) with total FAA, NH_3_ and propionic acid (data not shown). 

## Discussion

Increasing evidences indicate that environmental factors, including diet and gastrointestinal agents (e.g., microbiota), may play an important role in the autism spectrum disorders (ASD) [61]. Although variations on the composition of the microbiota were described for ASD patients compared to healthy individuals, the trend and importance are not already defined [14,33,35,59]. Overall, the microbiota of neurotypical sibling of AD was between the values of AD and HC children [11,34,35]. In another study [59], no significant differences were found between AD children and their neurotypical sibling HC groups based on 16S rDNA data [59]. 

This study showed that the main bacterial phyla (Firmicutes, Bacteroidetes, Fusobacteria and Verrucomicrobia) significantly differed in the fecal microbiota of PDD-NOS, AD and their sibling HC children. A shift at phylum level towards a higher proportion of Bacteroidetes and a lower level of Firmicutes was previously reported for fecal samples of AD children [35]. Compared to 16S-rDNA (total bacteria), the highest significant differences between the fecal microbiota of the three groups (PDD-NOS, AD and HC children) were found using 16S-rRNA data (metabolically active bacteria). Previously, it was shown that the active human gut microbiota differs from the total microbiota [62]. According to Gondalia et al. [59], PCA and Permutation analyses showed that PDD-NOS, AD and their sibling HC children were differentiable only based on 16S rRNA data. This result could be related to the environmental factors (e.g., diet) that mainly affected the total fecal microbiota [61]. *Faecalibacterium* and *Ruminococcus*, the main genera of Ruminococcaceae, were present at the highest levels in fecal samples of PDD-NOS and HC children. *F. prausnitzii*, which synthesizes short-chain fatty acids (SCFA) with anti-inflammatory properties, it was shown to decrease in Crohn disease patients [63]. Compared to AD children, the genus *Ruminococcus* increased in the fecal samples of HC [35]. With a few exceptions (e.g., *C. barletti*) [12], this study showed that the identified Clostridiaceae species were at the highest levels in AD children. Clostridiaceae are the one of the main bacterial group, which synthesizes some metabolic products (e.g., phenols, p-cresol, certain indole derivatives) that are potentially toxic for humans. It was also hypothesized that *Clostridium* species were associated with AD symptomology [33–35] and that the spore forming property of Clostridia is one of the main concern for the reoccurrence of autistic symptoms after oral vancomycin treatments [9]. Nevertheless, the association between the prevalence of *Clostridium* species and AD is still debated [59]. Compared to HC, also the composition of Lachnospiraceae family (*Roseburia, Dorea, Coprococcus* and *Lachnospira*) differed in the fecal samples of PDD-NOS and, especially, AD children. Species of the *Roseburia* and *Coprococcus* genera were described as capable to degrade starch and ferment other carbohydrates to synthesizing SCFA [64]. The composition of lactic acid bacteria (*Enterococcus*, *Lactobacillus* and *Streptococcus* genera) differed between AD and HC children [3,38]. The *Enterococcus* genus was found at the highest level in fecal samples of PDD-NOS children. In agreement with a previous report [35], the level of some Bacteroidetes genera (*Bacteroides*, *Barnesiella, Odoribacter* and *Parabacteroides*) and some *Prevotella* and *Alistipes* species were almost the highest in AD children. Previously, it was hypothesized that *Bacteroides* species produce propionic acid and other SCFA. Propionic acid showed neurobiological effects in rats [61]. Besides, *Bacteroides fragilis* group synthesizes lipopolysaccharide (LPS), an important bacterial virulence factor [35]. Usually, increased numbers of Bacteroidetes are found in fecal samples of children affected by GI inflammatory diseases and AD [35]. This study also showed that almost all identified Sutterellaceae (*Parasutterella* genus) and Enterobacteriaceae (e.g., *Proteus*, *Shigella*) were at higher levels in fecal samples of AD children compared to PDD-NOS and HC. The content of *Bifidobacterium* species decreased in fecal samples of AD. *Bifidobacterium* species were demonstrated to have species- and strain-specific influence on immunity [65]. Various species of *Bifidobacterium* synthesize exopolysaccharides, which act as fermentable substrates for human intestinal bacteria [66]. According to Finegold et al. [35], *Akkermansia muciniphila*, a novel mucin-degrading bacterium, was found at the highest level in AD children.

As determined by culture-dependent methods, cell densities of the main fecal microbial groups differed among fecal samples of PDD-NOS, AD and HC children. According to 16S rRNA data, the highest levels of presumptive *Clostridium*, *Bacteroides*, *Porphyromonas* and *Prevotella* and Enterobacteria were found in fecal samples of PDD-NOS and, especially AD children. *Enterococcus* and *Bifidobacterium* decreased in PDD-NOS and, especially AD children. 

The colonic fermentative activity is regulated by the amount and type of substrate that enters the colon. Nevertheless, the composition of the microbiota also affected the overall metabolome [67]. Low-molecular-weight metabolites produced by intestinal microbes play a direct role in health and disease. A very few studies considered the metabolome of fecal samples from AD and no reports are available for PDD-NOS. Previously, it was shown that serum and urine samples of AD patients had an altered level of FAA [42]. This study also showed that fecal samples of PDD-NOS and AD had an altered level of FAA. According to the level of dominant proteolytic genera (e.g., *Clostridium* and *Bacteroides*) [68], the amount of FAA increased in PDD-NOS and, especially, AD children. Many genera of Lachnospiraceae, which were found at high levels in AD children (e.g., *Roseburia* and *Dorea*), have a very poor capacity to degrade FAA [69]. On the contrary, *C. bartlettii* that shows high catabolic activity towards FAA [69] was found at high level in fecal samples of HC children. In agreement with these previous findings, this study showed a dysregulated metabolism of FAA for PDD-NOS and AD children. Some FAA, in particular Glu, also act as neurotransmitters in the central nervous system (CNS). Excess of Glu leads to neuronal cell death and Glu has an important role in the pathophysiology of some neuropsychiatric disorders [70]. Given the major role of Glu in brain development, it was hypothesized that glutamatergic neurotransmission is involved in ASD [42]. According to this hypothesis, Glu was found at the highest level in the fecal samples of AD children. 

Overall, phenol compounds (e.g., phenol, 4-(1,1-dimethylethyl)-phenol, p-cresol) increased in the feces of PDD-NOS and, especially, AD. Phenols derive from polyphenols, which are mainly associated with the consumption of vegetables and fruits. However, some phenol compounds were previously found related to specific bacteria (e.g., *Clostridium difficile*, *F. praunsnitzii*, *Bifidobacterium*) [67]. Complex polyphenols are metabolized via the gut microbiota into a few and simpler phenol compounds. These latter are further transformed at the liver level and released in the urines [71]. A hypothesis for the accumulation of polyphenols in the feces of AD children could be the low level of uridine-diphosphate-glucoronosyl-transferase activity in the liver [72]. Remarkably, the concentration of p-cresol increased in AD and PDD-NOS compared to HC children. The postnatal exposure to abnormal concentrations of p-cresol and/or p-cresylsulfate was considered as a pathoplastic contributor to the severity of behavioral abnormalities and cognitive impairment in ADS children [41]. While some ethyl esters were significantly associated with the feces of HC, methyl esters were mainly related to AD samples, which suggested a higher presence of methanol at the gut level. Methanol is a metabolic product of diet pectins, generally produced by pectinolytic *Clostridium* spp. Methanol is rarely found as free alcohol in the gut. In general, free alcohols and endogenous SCFA are metabolized into fatty acid esters in liver, pancreas and intestine [73]. At the intestinal site, esterification of alcohols by colonic bacteria (e.g., *Clostridium*) is regarded as a microbial strategy to remove or trap toxic molecules, including methanol. Sulphur compounds also differed within the three groups. Carbon disulfide was at the highest level in AD children. It may be synthesized through carbonation of hydrogen sulphide, as a detoxification mechanism exerted by colonic bacteria. In agreement with the previous report [3], AD children showed significantly lower levels of total SCFA compared to fecal samples of HC. The only exception was found for propionic acid, which is probably involved in the autism [61]. SCFA was mainly produced by clostridial clusters IV and XIVa of Firmicutes including species of *Roseburia*, *Faecalibacterium* and *Coprococcus*) (67). In this study, a connection was found between the concentration of SCFA and the level of *Faecalibacterium, Ruminococcus* and *Bifidobacterium*. SCFA represent the main fuel for colonocytes, and they are involved in water and electrolyte absorption by colon mucosa [74,75]. Indole and 3-methylindole increased in PDD-NOS and AD children. Indole is a microbial metabolite of tryptophane produced by several commensal bacteria that colonize the human GI tract [41]. Bansal et al. [76] hypothesized that indole is an inter kingdom signal in intestinal epithelial cells and play a strengthening effect on host cell-barrier properties. Moreover, it is a critical precursor for the biosynthesis of physiologically important molecules such as serotonin and melatonin. Serotonin, a monoamine neurotransmitter, is not formed by bacterial action, but extensive activities of bacteria related to other indole derivatives (e.g., 3-methylindole) is interesting particularly in the light of serotonin-function in social interaction, mood disorders, obsessive-compulsive activity [77].

Compared to HC, ASD children had an altered fecal microbiota and VOCs, which were partially different between PDD-NOS and AD. This study showed that the fecal microbiota, FAA and VOCs of PDD-NOS were more similar to HC than those of AD. The main biological significance of this work was related to the decreased level of some healthy promoting bacteria (e.g., *Bifidobacterium*) and metabolites (e.g., FAA, SCFA) in PDD-NOS and, especially, in AD children. Dietary implementation with prebiotics and probiotics could be a useful tool to restore some microbial gaps (e.g., *Bifidobacterium*). Combining the results of this work with those from previous reports [3,35,41], it seemed to emerge that microbial indices (e.g., *Clostridium*) and levels of some metabolites (e.g., Glu, p-cresol) might be signatures for PDD-NOS and, especially, AD children. 

## Supporting Information

Figure S1 **Comparison of total and active bacterial phyla found in feces of children.** Bacterial phyla distribution (%) found in fecal samples of Pervasive Developmental Disorder Not Otherwise Specified (PDD-NOS), autistic (AD) and healthy (HC) children. The X-axis represents the proportion of phyla from total (16S rDNA) and active (16S rRNA) bacteria.
(TIF)

Figure S2 **Principal component analysis (PCA) of total bacteria genera found in feces of children.** Score plot (A) and loading plot (B) of the three principal components (PC) after PCA of the total bacterial genera information (16S rDNA) for Pervasive Developmental Disorder Not Otherwise Specified (PDD-NOS), autistic (AD) and healthy (HC) children. 1-10, number of fecal samples for each group of children.
(TIF)

Table S1 **Volatile organic compounds (VOCs).** Concentration (ppm) of VOCs of fecal samples of Pervasive Developmental Disorder Not Otherwise Specified (PDD-NOS), autistic (AD) and healthy (HC) children.
(DOC)
