# Supplementary figures and images for: Fecal Microbiota and Metabolome of Children with Autism and Pervasive Developmental Disorder Not Otherwise Specified
Source: PLoS One. 2013 Oct 9;8(10):e76993. doi: 10.1371/journal.pone.0076993 (PMC3793965; doi:10.1371/journal.pone.0076993)

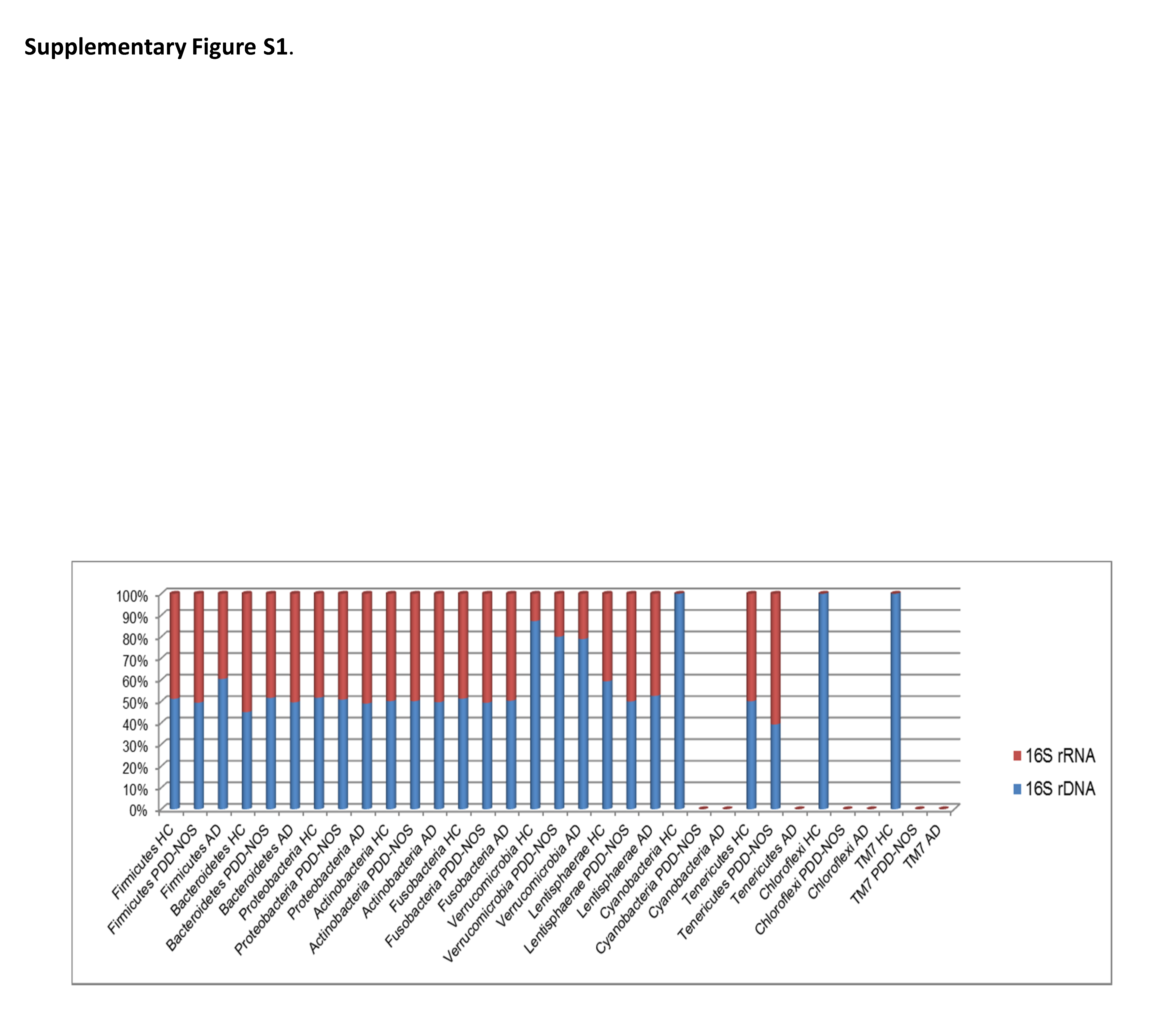

Supplement: Figure S1 — Comparison of total and active bacterial phyla found in feces of children. Bacterial phyla distribution (%) found in fecal samples of Pervasive Developmental Disorder Not Otherwise Specified (PDD-NOS), autistic (AD) and healthy (HC) children. The X-axis represents the proportion of phyla from total (16S rDNA) and active (16S rRNA) bacteria. (TIF) [file pone.0076993.s001.tif]

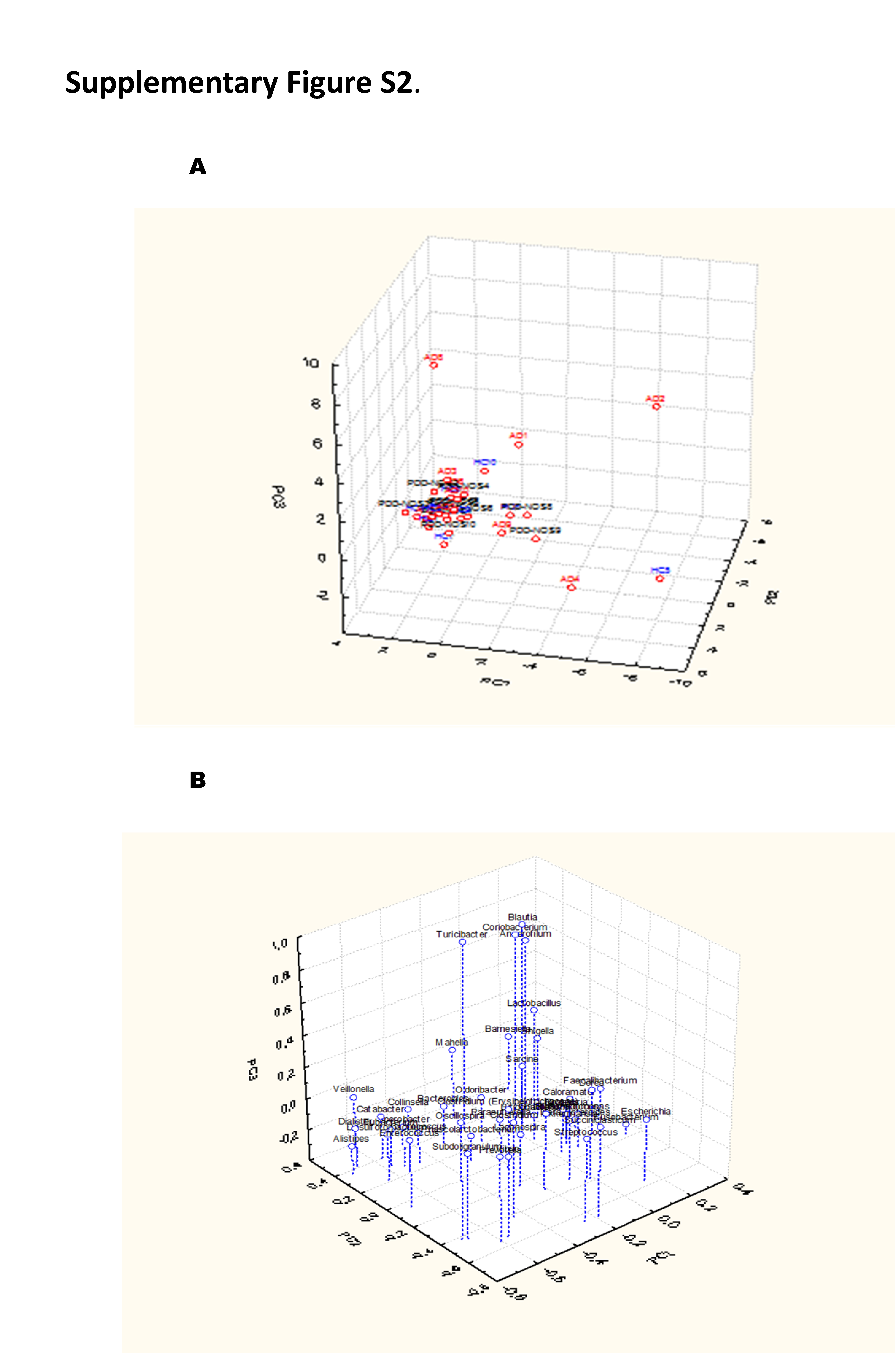

Supplement: Figure S2 — Principal component analysis (PCA) of total bacteria genera found in feces of children. Score plot (A) and loading plot (B) of the three principal components (PC) after PCA of the total bacterial genera information (16S rDNA) for Pervasive Developmental Disorder Not Otherwise Specified (PDD-NOS), autistic (AD) and healthy (HC) children. 1-10, number of fecal samples for each group of children. (TIF) [file pone.0076993.s002.tif]
