# Supplementary material for: Fecal Microbiota and Metabolome of Children with Autism and Pervasive Developmental Disorder Not Otherwise Specified
Source: PLoS One. 2013 Oct 9;8(10):e76993. doi: 10.1371/journal.pone.0076993 (PMC3793965; doi:10.1371/journal.pone.0076993)
Supplement: Table S1 — Volatile organic compounds (VOCs). Concentration (ppm) of VOCs of fecal samples of Pervasive Developmental Disorder Not Otherwise Specified (PDD-NOS), autistic (AD) and healthy (HC) children. (DOC) [file pone.0076993.s003.doc]

**Table S1**. Volatile organic compounds (VOCs). Concentration (ppm) of VOCs of fecal samples of Pervasive Developmental Disorder Not Otherwise Specified (PDD-NOS), autistic (AD) and healthy (HC) children.

| **Chemical class** | **PDD-NOS** | | **AD** | | **HC** | |
| --- | --- | --- | --- | --- | --- | --- |
| Average | | Range | Average | Range | Average | Range |
| Alcohols | | | | | | |
| Ethanol | 2.56^b^ | 0.45-12.35 | 1.49^c^ | 0.38-4.93 | 4.43^a^ | 0.26-44.66 |
| 3-Methyl-1-pentanol | 0.36^a^ | 0.04-1.15 | 0.24^b^ | 0-0.58 | 0.24^b^ | 0-1.01 |
| 3,7-Dimethyl-2,6-octadien-1-ol | 1.90^a^ | 0.02-4.79 | 1.71^b^ | 0.03-10.72 | 1.42^c^ | 0.01-12.21 |
| 1-Pentanol | 0.54^a^ | 0.09-2.46 | 0.45^b^ | 0.07-1.70 | 0.61^a^ | 0.07-2.87 |
| 3-Methyl-2-butanol | 0^a^ | 0 | 0^a^ | 0 | 0.01^a^ | 0-0.12 |
| Heptanol | 0.01^a^ | 0-0.03 | 0.02^a^ | 0-0.17 | 0.01^a^ | 0-0.08 |
| 2-Propyl-1-pentanol | 0.02^b^ | 0.01-0.08 | 0.01^c^ | 0.00-0.04 | 0.03^a^ | 0.01-0.16 |
| 1-[2-Methyl-3-(methylthio) allyl]cyclohex-2-enol | 0.80^a^ | 0.45-0.98 | 0.52^b^ | 0-0.98 | 0.76^a^ | 0.36-1.58 |
| 4-(1,1,3,3-tetramethylbutyl)-Phenol | 0.58^a^ | 0.19-1.68 | 0.58^a^ | 0.18-1.35 | 0.47^b^ | 0.19-0.79 |
| 1-Octanol | 0.01^a^ | 0-0.01 | 0.02^a^ | 0-0.11 | 0.01^a^ | 0-0.02 |
| Phenylethyl alcohol | 0.01^a^ | 0-0.02 | 0.01^a^ | 0-0.04 | 0.01^a^ | 0-0.02 |
| Phenol | 0.20^a^ | 0.01-1.01 | 0.16^a^ | 0.02-1.01 | 0.10^b^ | 0.01-0.83 |
| 4-(1,1-Dimethylethyl)-phenol | 0.18^a^ | 0.06-0.18 | 0.16^a^ | 0.04-0.14 | 0.09^b^ | 0.05-0.13 |
| p-Cresol | 5.23^a^ | 0.48-11.83 | 4.67^b^ | 0.17-21.54 | 3.11^c^ | 0.19-8.34 |

**Table 1S** continued

| **Chemical class** | **PDD-NOS** | | **AD** | | **HC** | |
| --- | --- | --- | --- | --- | --- | --- |
| Average | | Range | Average | Range | Average | Range |
| Total alcohols | 12.40^a^ | / | 10.25^c^ | / | 11.19^b^ | / |
| Aldehydes | | | | | | |
| Octanal | 0.17^a^ | 0-0.64 | 0.02^c^ | 0-0.07 | 0.12^b^ | 0-0.78 |
| Nonanal | 0.04^a^ | 0.02-0.08 | 0.04^a^ | 0.02-0.06 | 0.03^b^ | 0.01-0.08 |
| Benzeneacetaldehyde | 0.04^a^ | 0-0.11 | 0.04^a^ | 0.01-0.08 | 0.03^b^ | 0.01-0.09 |
| Benzaldehyde | 0.11^a^ | 0.02-0.32 | 0.10^a^ | 0.01-0.27 | 0.06^b^ | 0.02-0.15 |
| Total aldehydes | 0.37^a^ | / | 0.20^c^ | / | 0.24^b^ | / |
| Esters | | | | | | |
| Acetic acid methyl ester | 2.00^b^ | 0.93-2.52 | 2.20^a^ | 0.60-4.25 | 1.87^b^ | 0.43-5.84 |
| Acetic acid ethyl ester | 0.64^b^ | 0.04-2.14 | 0.40^c^ | 0.07-0.79 | 0.82^a^ | 0.06-5.43 |
| Propionic acid ethyl ester | 2.06^a^ | 0.10-6.80 | 0.70^c^ | 0.22-2.26 | 1.37^b^ | 0.09-7.45 |
| Propionic acid 2-methylethyl ester | 0.02^a^ | 0-0.06 | 0.01^a^ | 0-0.02 | 0.02^a^ | 0-0.09 |
| Butanoic acid methyl ester | 6.54^a^ | 1.56-14.84 | 5.76^b^ | 1.73-12.71 | 5.30^c^ | 1.75-15.22 |
| Butanoic acid ethyl ester | 3.11^a^ | 0.08-8.22 | 1.11^c^ | 0.25-2.18 | 2.87^b^ | 0.06-16.63 |
| Butanoic acid 3-methylethyl ester | 0.02^a^ | 0-0.13 | 0.01^a^ | 0-0.02 | 0.01^a^ | 0-0.06 |
| Acetic acid butyl ester | 0.19^a^ | 0-0.41 | 0.06^c^ | 0-0.33 | 0.13^b^ | 0-0.79 |

**Table 1S** continued

| **Chemical class** | **PDD-NOS** | | **AD** | | **HC** | |
| --- | --- | --- | --- | --- | --- | --- |
| Average | | Range | Average | Range | Average | Range |
| Pentanoic acid methyl ester | 0.41^a^ | 0.02-2.32 | 0.39^a^ | 0.02-1.89 | 0.25^b^ | 0.01-0.89 |
| Butanoic acid propyl ester | 1.80^a^ | 0.07-9.25 | 0.64^c^ | 0.10-1.55 | 1.70^b^ | 0.07-8.48 |
| Pentanoic acid ethyl ester | 0.76^a^ | 0.01-3.55 | 0.28^c^ | 0.01-1.48 | 0.71^b^ | 0.02-7.58 |
| Heptanoic acid, 1,1-dimethylethyl ester | 0.03^b^ | 0.00-0.07 | 0.03^b^ | 0.00-0.08 | 0.05^a^ | 0.01-0.08 |
| Pentanoic acid 3-methylmethyl ester | 0.02^b^ | 0-0.23 | 0.03^a^ | 0-0.08 | 0.04^a^ | 0-0.15 |
| Butanoic acid butyl ester | 2.59^a^ | 0.15-18.24 | 0.69^c^ | 0.12-2.17 | 1.66^b^ | 0.09-12.12 |
| Butanoic acid hexyl ester | 0.39^a^ | 0.01-1.87 | 0.15^b^ | 0.01-0.89 | 0.41^a^ | 0-4.74 |
| Hexanoic acid ethyl ester | 0.05^b^ | 0-0.35 | 0.02^c^ | 0-0.18 | 0.11^a^ | 0-1.88 |
| Butanoic acid 3-methylbutyl ester | 0.04^a^ | 0-0.15 | 0.04^a^ | 0-0.19 | 0.03^b^ | 0-0.17 |
| Acetic acid hexyl ester | 0^a^ | 0 | 0.01^a^ | 0-0.04 | 0^a^ | 0 |
| Heptanoic acid methyl ester | 0.01^a^ | 0-0.04 | 0^a^ | 0 | 0^a^ | 0 |
| Hexanoic acid propyl ester | 0.01^a^ | 0-0.03 | 0.01^a^ | 0-0.06 | 0.01^a^ | 0-0.08 |
| Pentanoic acid hexyl ester | 0.03^a^ | 0.01-0.18 | 0.03^a^ | 0-0.17 | 0.02^b^ | 0-0.14 |
| Heptanoic acid ethyl ester | 0.02^b^ | 0-0.19 | 0.01^b^ | 0-0.09 | 0.04^a^ | 0-0.70 |
| Propionic acid hexyl ester | 0.01^a^ | 0-0.03 | 0.01^a^ | 0-0.06 | 0.01^a^ | 0.01-0.03 |
| Hexanoic acid 2-methylpropyl ester | 0^a^ | 0 | 0.01^a^ | 0-0.06 | 0^a^ | 0 |
| Heptanoic acid 1-methylethyl ester | 0.05^a^ | 0.03-0.06 | 0.05^a^ | 0.03-0.14 | 0.04^b^ | 0.02-0.07 |

**Table 1S** continued

| **Chemical class** | **PDD-NOS** | | **AD** | | **HC** | |
| --- | --- | --- | --- | --- | --- | --- |
| Average | | Range | Average | Range | Average | Range |
| Benzoic acid hexadecyl ester | 0.10^a^ | 0.05-0.08 | 0.10^a^ | 0.04-0.08 | 0.06^b^ | 0.04-0.16 |
| Phthalic acid diethyl ester | 0^a^ | 0 | 0.01^a^ | 0-0.01 | 0.016^a^ | 0-0.03 |
| Phthalic acid methyl neopentyl ester | 0.20^a^ | 0.11-0.25 | 0.19^a^ | 0.07-0.23 | 0.16^b^ | 0.10-0.26 |
| Total esters | 21.10^a^ | / | 12.95^c^ | / | 17.70^b^ | / |
| Sulphur compounds | | | | | | |
| Carbon disulphide | 64.09^c^ | 37.58-84.57 | 64.90^a^ | 41.63-81.25 | 64.75^b^ | 10.76-128.68 |
| Dimethyl disulphide | 2.44^b^ | 0.27-8.87 | 2.08^c^ | 0.03-6.88 | 3.07^a^ | 0.08-7.79 |
| Dimethyl trisulphide | 0.04^b^ | 0.01-0.13 | 0.05^b^ | 0.02-0.11 | 0.06^a^ | 0.01-0.16 |
| Total sulphur compounds | 66.57^c^ | / | 67.03^b^ | / | 67.88^a^ | / |
| Hydrocarbons | | | | | | |
| Octane | 0.01^a^ | 0-0.02 | 0.02^a^ | 0.01-0.04 | 0.02^a^ | 0.01-0.07 |
| Benzene | 2.37^a^ | 0.75-3.91 | 2.01^c^ | 0.45-5.74 | 2.18^b^ | 0.54-5.94 |
| Trichloromethane | 1.19^b^ | 0.23-2.99 | 1.15^b^ | 0.26-2.58 | 1.44^a^ | 0.27-7.15 |
| Toluene | 1.15^a^ | 0.45-2.16 | 1.24^a^ | 0.10-3.39 | 1.09^b^ | 0.13-3.66 |
| O-xylene | 0.28^a^ | 0.11-0.58 | 0.28^a^ | 0.09-0.70 | 0.24^b^ | 0.05-0.52 |
| Isopentyl alcohol, formate | 0.04^a^ | 0.01-0.10 | 0.02^c^ | 0.01-0.04 | 0.03^b^ | 0.01-0.16 |
| 4-Hydroxy-3-methyl-2-butanonene | 0.02^a^ | 0-0.03 | 0.02^a^ | 0-0.07 | 0.01^a^ | 0-0.04 |
| 1-Methyl-2-(1-methylethyl)-benzene | 0.53^b^ | 0.03-1.71 | 1.12^a^ | 0.02-4.93 | 0.46^c^ | 0.02-3.60 |

**Table 1S** continued

| **Chemical class** | **PDD-NOS** | | **AD** | | **HC** | |
| --- | --- | --- | --- | --- | --- | --- |
| Average | | Range | Average | Range | Average | Range |
| 2,2,4,6,6-Pentamethyl-3-heptene | 1.01^a^ | 0.49-1.98 | 0.95^a^ | 0.46-1.58 | 0.88^b^ | 0.41-1.66 |
| 6-Methyl-1-heptene | 0.47^a^ | 0.11-1.67 | 0.37^b^ | 0.07-1.75 | 0.34^c^ | 0.10-1.60 |
| 1,4-Bis-(1,1-dimethylethyl)-benzene | 2.01^b^ | 0.60-3.33 | 2.70^a^ | 0.63-6.72 | 1.74^c^ | 0.61-4.42 |
| 2-Pentyl-thiophene | 0.01^a^ | 0.01-0.01 | 0.01^a^ | 0-0.01 | 0.01^a^ | 0-0.01 |
| Total hydrocarbons | 9.09^b^ | / | 9.89^a^ | / | 8.44^c^ | / |
| Ketones | | | | | | |
| 2-Butanone | 5.59^b^ | 1.19-19.79 | 5.21^b^ | 0.89-14.35 | 10.59^a^ | 1.02-90.26 |
| Methyl isobutyl ketone | 0.10^a^ | 0.06-0.11 | 0.11^a^ | 0.08-0.13 | 0.05^b^ | 0.03-0.14 |
| 3-Hexanone | 0.07^b^ | 0.01-0.28 | 0.05^b^ | 0-0.24 | 0.10^a^ | 0-0.50 |
| 4-Heptanone | 0.03^b^ | 0.01-0.21 | 0.03^b^ | 0.01-0.06 | 0.05^a^ | 0.01-0.53 |
| 2,6-Dimethyl-4-heptanone | 1.46^a^ | 0.76-2.42 | 1.49^a^ | 0.93-2.34 | 1.41^b^ | 0.93-2.20 |
| 1-Phenyl-2-hexanone | 0.09^b^ | 0.04-0.13 | 0.32^a^ | 0.05-1.38 | 0.06^c^ | 0.03-0.14 |
| Total ketones | 7.34^b^ | / | 7.22^b^ | / | 12.25^a^ | / |
| Short and medium chain fatty acids | | | | | | |
| Acetic acid | 0.38^a^ | 0.07-1.17 | 0.44^a^ | 0.06-1.14 | 0.33^b^ | 0.05-1.33 |
| Propionic acid | 0.35^a^ | 0.03-1.15 | 0.37^a^ | 0.06-0.86 | 0.31^b^ | 0.01-1.00 |
| Butanoic acid | 2.92^b^ | 0.21-10.08 | 1.77^c^ | 0-4.14 | 2.62^a^ | 0.04-16.80 |
| 3-Methylbutanoic acid | 0.04^b^ | 0-0.09 | 0.04^b^ | 0.01-0.11 | 0.05^a^ | 0-0.21 |

**Table 1S** continued

| **Chemical class** | **HC** | | **AD** | | **PDD-NOS** | |
| --- | --- | --- | --- | --- | --- | --- |
| Average | | Range | Average | Range | Average | Range |
| 2-Methylbutanoic acid | 0.14^b^ | 0.02-0.33 | 0.14^b^ | 0.03-0.26 | 0.16^a^ | 0.02-0.65 |
| Pentanoic acid | 0.53^b^ | 0.06-2.05 | 0.45^b^ | 0.05-1.55 | 0.62^a^ | 0.03-2.65 |
| Hexanoic acid | 0.04^b^ | 0-0.19 | 0.04^b^ | 0-0.27 | 0.07^a^ | 0-0.50 |
| Heptanoic acid | 0.01^a^ | 0-0.04 | 0.01^a^ | 0-0.05 | 0.01^a^ | 0-0.08 |
| Octanoic acid | 0^a^ | 0 | 0.01^a^ | 0-0.04 | 0^a^ | 0 |
| Total short and medium chain fatty acids | 4.41^a^ | / | 3.27^c^ | / | 4.17^b^ | / |
| Terpenes | | | | | | |
| (-)-Cis-Carene | 0.87^a^ | 0.09-1.60 | 0.76^b^ | 0.29-1.57 | 0.71^c^ | 0.05-1.32 |
| Limonene | 0.32^a^ | 0-1.09 | 0.09^c^ | 0.01-1.29 | 0.21^b^ | 0-1.82 |
| Copaene | 0^a^ | 0 | 0.01^a^ | 0-0.02 | 0.01^a^ | 0-0.03 |
| Menthol | 0.02^a^ | 0-0.12 | 0^a^ | 0 | 0.02^a^ | 0-0.13 |
| Total terpenes | 1.22^a^ | / | 0.86^b^ | / | 0.94^b^ | / |
| Indoles | | | | | | |
| Indole | 0.74^a^ | 0.15-2.25 | 0.46^b^ | 0.11-1.08 | 0.31^c^ | 0.10-1.36 |
| 3-Methylindole | 0.24^b^ | 0.01-0.87 | 0.83^a^ | 0.01-7.93 | 0.14^c^ | 0.01-1.08 |
| Total indoles | 0.98^b^ | / | 1.29^a^ | / | 0.45^c^ | / |
| Others | | | | | | |
| Furanone A | 2.76^a^ | 1.72-3.31 | 2.49^b^ | 1.57-2.94 | 2.33^b^ | 1.02-6.02 |

Data are the means of three independent experiments (n = 3) for each children. ^a–c^Values within a row with different superscript letters are significantly different (p < 0.05).
